# Epigenetic loss of the transfer RNA-modifying enzyme TYW2 induces ribosome frameshifts in colon cancer

**DOI:** 10.1073/pnas.2003358117

**Published:** 2020-08-10

**Authors:** Margalida Rosselló-Tortella, Pere Llinàs-Arias, Yuriko Sakaguchi, Kenjyo Miyauchi, Veronica Davalos, Fernando Setien, Maria E. Calleja-Cervantes, David Piñeyro, Jesús Martínez-Gómez, Sonia Guil, Ricky Joshi, Alberto Villanueva, Tsutomu Suzuki, Manel Esteller

**Affiliations:** ^a^Cancer Epigenetics Group, Josep Carreras Leukaemia Research Institute, 08916, Badalona, Barcelona, Catalonia, Spain;; ^b^Germans Trias i Pujol Health Science Research Institute, 08916, Badalona, Barcelona, Catalonia, Spain;; ^c^Department of Chemistry and Biotechnology, Graduate School of Engineering, University of Tokyo, 113-8656 Tokyo, Japan;; ^d^Centro de Investigacion Biomedica en Red Cancer, 28029 Madrid, Spain;; ^e^Translational Research Laboratory, Catalan Institute of Oncology, Bellvitge Biomedical Research Institute, 08908, L'Hospitalet de Llobregat, Barcelona, Catalonia, Spain;; ^f^Institucio Catalana de Recerca i Estudis Avançats, 08010 Barcelona, Catalonia, Spain;; ^g^Physiological Sciences Department, School of Medicine and Health Sciences, University of Barcelona, 08907 Barcelona, Catalonia, Spain

**Keywords:** epigenetics, transfer RNA, cancer

## Abstract

Transfer RNA (tRNA) activity is tightly regulated to provide a physiological protein translation, and tRNA chemical modifications control its function in a complex with ribosomes and messenger RNAs (mRNAs). In this regard, the correct hypermodification of position G37 of phenylalanine-tRNA, adjacent to the anticodon, is critical to prevent ribosome frameshifting events. Here we report that the tRNA-yW Synthesizing Protein 2 (TYW2) undergoes promoter hypermethylation-associated transcriptional silencing in human cancer, particularly in colorectal tumors. The epigenetic loss of TYW2 induces guanosine hypomodification in phenylalanine-tRNA, an increase in −1 ribosome frameshift events, and down-regulation of transcripts by mRNA decay, such as of the key cancer gene ROBO1. Importantly, TYW2 epigenetic inactivation is linked to poor overall survival in patients with early-stage colorectal cancer, a finding that could be related to the observed acquisition of enhanced migration properties and epithelial-to-mesenchymal features in the colon cancer cells that harbor TYW2 DNA methylation-associated loss. These findings provide an illustrative example of how epigenetic changes can modify the epitranscriptome and further support a role for tRNA modifications in cancer biology.

## Significance

Defects in transfer RNA (tRNA) modifications occur in human pathologies such as cancer; however, how these alterations contribute to the disease is poorly understood. One example is the tumor-specific hypomodification of position 37 of tRNA^Phe^, which was first described 45 y ago, although its cause and consequences have remained unknown. Here we report that the tRNA^Phe^ hypomodification is due to promoter CpG island hypermethylation-associated transcriptional silencing of TYW2, a key enzyme in the synthesis of wybutosine derivatives. Furthermore, epigenetic loss of TYW2 in transformed cells provokes hypomodified tRNA^Phe^-mediated ribosome frameshifting, dysregulating mRNA abundance via nonsense-mediated decay. Importantly, TYW2 silencing in cancer cells confers enhanced migration and epithelial-to-mesenchymal features that are associated in early-stage colorectal cancer patients with poor clinical outcome.

Coding and noncoding RNA molecules harbor chemically modified nucleosides that together constitute the so-called “epitranscriptome” (1, 2). These chemical modifications are particularly abundant in transfer RNA molecules (tRNAs), with more than 50 different modifications described in eukaryotic tRNAs (3–6). Modified bases in tRNA are critical for its translational function at multiple levels, including amino acid loading, wobbling or translation efficiency, and fidelity, among others (5, 7). tRNA modifications also serve as a translational regulation mechanism under stress conditions (4). Defects in tRNA modifications or tRNA modifier enzymes are present in various human pathologies, including neurologic disorders, mitochondrial diseases, and cancers (8–10).

The causes and roles of the tRNA modification changes observed in cancer are poorly understood. One example of chemical modifications found in tRNA that has not been studied in depth in the context of transformed cells is the existence of wybutosine (yW)-derived nucleosides in position G37, adjacent to the anticodon of phenylalanine tRNA (tRNA^Phe^). Highly modified purines in position 37 of various tRNAs are key for maintaining the ribosome reading frame and ensuring translation fidelity. Concretely, yW, first described in 1968 and originally called Y-base (11), prevents −1 programmed ribosome frameshifting (PRF) events (12) as a result of a proper stabilization of the codon-anticodon pairing through base-stacking interaction (13). Ribosome frameshifts occur in sequences with a specific structure comprising a slippery heptamer followed by a complex secondary structure (14). The loss of yW enhances −1 PRF events in viral sequences (12, 15), which is essential for the correct expression of viral proteins. Ribosome frameshifting also occurs in messenger RNA (mRNA). When ribosomes slip and alter the reading frame, a premature stop codon usually appears, and the mRNA is degraded via nonsense-mediated decay (NMD) (16). Therefore, PRF events are postulated to be an additional layer of posttranscriptional regulation of mRNA abundance.

An eye-opening stream of data indicating a role for the hypermodification of position G37 in cancer occurred in the late 1970s, when various researchers highlighted in prominent publications the tumor-specific loss of the Y-base of tRNA^Phe^ and suggested that this phenomenon could provide a growth advantage to these cells (17–19). The underlying mechanism has remained unknown for the last 45 y, however. To solve this enigma, we wondered about the presence of cancer-specific defects in the six enzymes, TRMT5 (20) and TYW1–5 (21, 22), that act sequentially in humans to modify the original G37 nucleotide to finally obtain the hypermodified yW in its hydroxylated (OHyW) or peroxydated (o2yW) form. Since transcriptional silencing associated with promoter CpG island hypermethylation is a frequent mechanism of gene inactivation in transformed cells (23, 24), we interrogated the presence of this type of epigenetic inactivation in the genes encoding the enzymes responsible for yW derivative synthesis. We found that TYW2 undergoes tumor-specific silencing by promoter CpG island hypermethylation, preventing formation of the hypermodified forms of G37. Those cancer cells with the hypomodified guanosine were prone to ribosome frameshifting events. Most importantly, the epigenetic defect in *TYW2* was associated with poor clinical outcome of the studied colorectal cancer, an observation that can be linked to the acquisition of enhanced cellular migration features and epithelial-to-mesenchymal features on TYW2 loss.

## Results

### Promoter CpG Island Hypermethylation-Associated Transcriptional Silencing of the tRNA-Modifying Enzyme TYW2 in Colon Cancer.

**Fig. 1.**
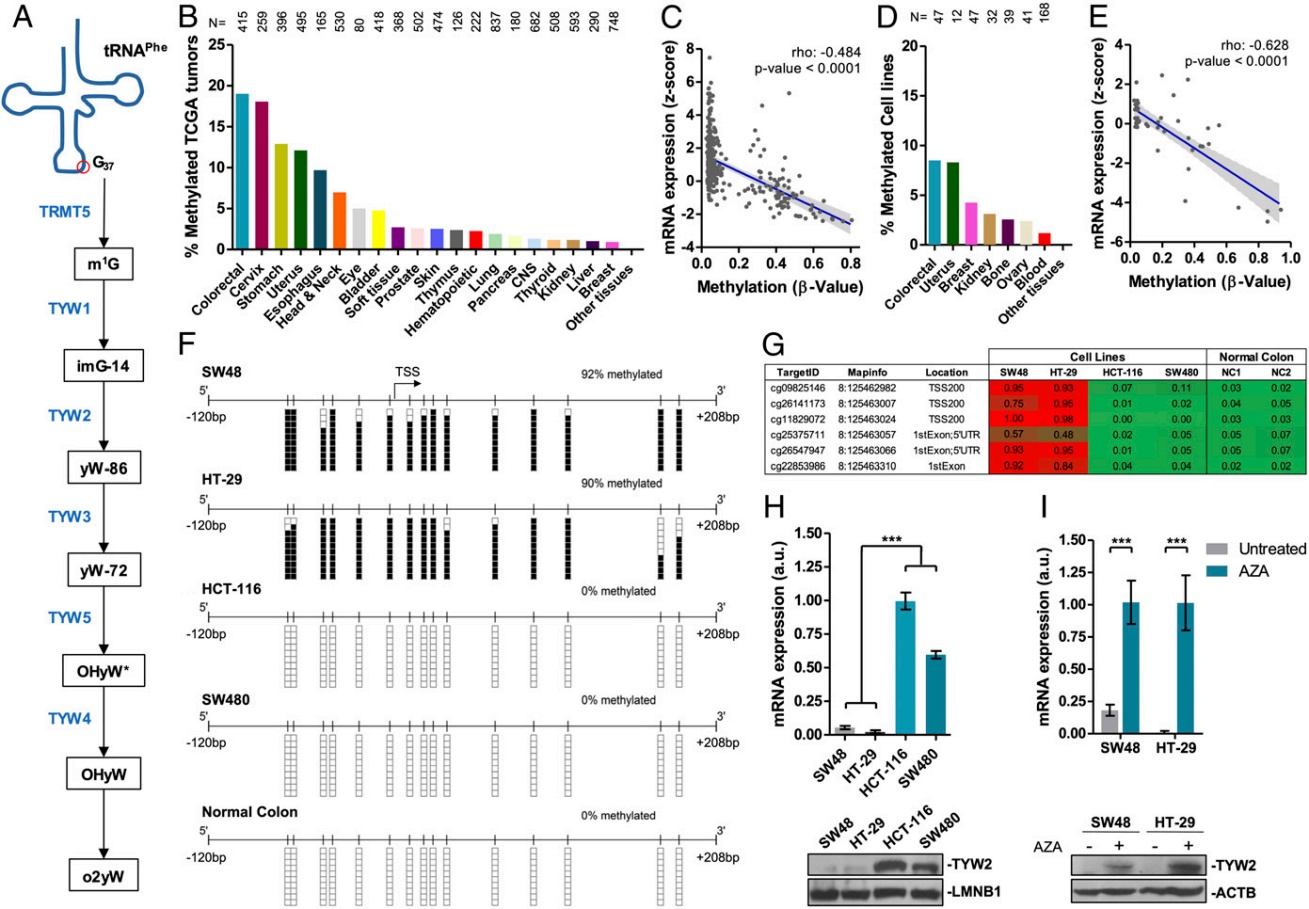
*TYW2* promoter CpG island hypermethylation-associated transcriptional silencing in primary colorectal tumors and cancer cell lines. (*A*) Schematic representation of wybutosine derivative synthesis at position G37 of human tRNA^Phe^. The enzymes catalyzing these reactions are shown in blue. Abbreviations for the modified G37 nucleoside are as follows: m^1^G, 1-methylguanosine; imG-14, 4-demethylwyosine; yW-86, 7-aminocarboxypropyl-demethylwyosine; yW-72, 7-aminocarboxypropylwyosine; OHyW*, undermodified hydroxywybutosine; OHyW, hydroxywybutosine; o2yW, peroxywybutosine. (*B*) Frequency of *TYW2* hypermethylation in primary tumors derived from TCGA according to cancer type. (*C*) The presence of TYW2 methylation is significantly associated with loss of expression of the *TYW2* transcript in primary colorectal tumors from TCGA. Spearman correlation, *P* < 0.001. (*D*) Frequency of *TYW2* hypermethylation in cancer cell lines derived from the Sanger panel according tumor type. (*E*) The presence of *TYW2* methylation is significantly associated with loss of expression of the *TYW2* transcript in colorectal cancer cell lines from the Sanger panel. Spearman correlation, *P* < 0.001. (*F*) Bisulfite genomic sequencing of *TYW2* promoter CpG island in colon cancer cell lines and in normal colon. CpG dinucleotides are represented as short vertical lines; the TSS is indicated with a black arrow. Single clones are shown for each sample. The methylation status of each CpG dinucleotide within the interrogated sequence is denoted with a black (methylated) or white (unmethylated) square. (*G*) DNA methylation profile of the *TYW2* promoter CpG island analyzed by the Infinium 450K DNA methylation array. Single CpG absolute methylation β-values are shown (0 to 1). Red, methylated; green, unmethylated. Data from four colon cancer cell lines and two normal colon samples are shown. (*H*) TYW2 expression levels in methylated (SW48 and HT-29) and unmethylated (HCT-116 and SW480) cancer cell lines determined by qRT-PCR (*Top*) and Western blot analysis (*Bottom*). The qRT-PCR data shown represent the mean ± SD of at least three biological replicates and were analyzed using the unpaired two-tailed Student’s *t* test. ***P < 0.001. (*I*) Recovery of TYW2 transcript (*Top*) and protein (*Bottom*) expression in hypermethylated colon cancer cell lines SW48 and HT-29 on use of the demethylating agent 5-aza-2’-deoxycytidine (AZA). The qRT-PCR data shown represent the mean ± SD of at least three biological replicates and were analyzed using the unpaired two-tailed Student’s *t* test. ***P < 0.001.

To identify epigenetic defects in the enzymes involved in generating the hypermodified guanosine nucleotide in the tRNA^Phe^, we studied the DNA methylation status of the 5′-end–associated CpG islands of *TRMT5*, *TYW1*, *TYW2*, *TYW3*, *TYW4*, and *TYW5* genes. These six human enzymes were identified as involved in the different chemical modifications that this nucleotide undergoes from its initial G37 form (Fig. 1*A*) (21, 22). Data mining of thousands of primary tumors corresponding to all cancer types available at The Cancer Genome Atlas (TCGA) (https://www.cancer.gov/about-nci/organization/ccg/research/structural-genomics/tcga) did not indicate the presence of *TRMT5*, *TYW1*, *TYW3*, *TYW4* or *TYW5* promoter hypermethylation events (*SI Appendix*, Fig. S1*A* and Dataset S1). In contrast, the *TYW2* promoter CpG island was methylated in 19.03% (79 of 415) of the primary colorectal carcinomas available in the TCGA (Fig. 1*B* and Dataset S1). Beyond colorectal tumors, the *TYW2* promoter CpG island was also commonly hypermethylated in cervical (17.37%; 45 of 259), gastric (12.88%; 51 of 396). and uterine (12.12%; 60 of 495) carcinomas (Fig. 1*B* and Dataset S1). For TYW2, as well as the other five tRNA-modifying enzymes studied here, the promoter CpG island was always unmethylated in all the normal human tissues studied (Dataset S2). Data mining of the TCGA RNA-sequencing (RNA-seq) data available in colorectal tumors showed that *TYW2* hypermethylation was associated with transcript down-regulation (Spearman rho = −0.47; *P* < 0.0001) (Fig. 1*C*). In this regard, TYW2 was the only G37 tRNA^Phe^- modifying enzyme showing RNA down-regulation in the TCGA dataset (*SI Appendix*, Fig. S2).

We also data mined the Sanger set of 1,001 human cancer cell lines, in which we had recently obtained the DNA methylation and expression profiles (25), and obtained results similar to those observed in the TCGA cohorts (*SI Appendix*, Fig. S1*B* and Dataset S3). In this regard, colorectal cancer cell lines were the most frequently *TYW2*-hypermethylated tumor type (Fig. 1*D* and Dataset S3), and *TYW2* hypermethylation was also associated with transcript down-regulation (Fig. 1*E*), mimicking the results observed in the TCGA cohorts. Thus, the higher frequency of aberrant *TYW2* DNA methylation-associated transcriptional silencing observed in colorectal cancer prompted us to focus our efforts on this tumor type.

We analyzed in more detail the link between *TYW2* promoter CpG island hypermethylation and transcriptional inactivation of the gene at the RNA and protein levels in colon cancer cell lines. We performed bisulfite genomic sequencing of multiple clones from SW48, HT-29, HCT-116, and SW480 cells using primers that encompassed the transcription start site (TSS)-associated CpG island. We observed that the 5′-end CpG island of *TYW2* in the SW48 and HT-29 cell lines was hypermethylated compared with normal colon mucosa (Fig. 1*F*), whereas the HCT-116 and SW480 cells were unmethylated (Fig. 1*F*). These results mimicked the DNA methylation patterns found by the microarray approach used in the Sanger cell line cohort (25) (Fig. 1*G*). Importantly, the *TYW2* hypermethylated colon cancer cell lines SW48 and HT-29 minimally expressed the TYW2 RNA transcript and protein, as determined by quantitative real-time PCR and Western blot analysis, respectively (Fig. 1*H*). In contrast, the unmethylated cell lines HCT-116 and SW480 expressed the TYW2 transcript and protein (Fig. 1*H*). The use of the inhibitor of DNA methylation 5-aza-2-deoxycytidine in the *TYW2* hypermethylated cell lines restored its expression at the RNA and protein levels (Fig. 1*I*), reinforcing the link between *TYW2* promoter hypermethylation and transcriptional silencing.

### TYW2 Epigenetic Silencing in Cancer Cells Mediates Loss of Hypermodified Guanosine in tRNA and Ribosomal Frameshifting.

**Fig. 2.**
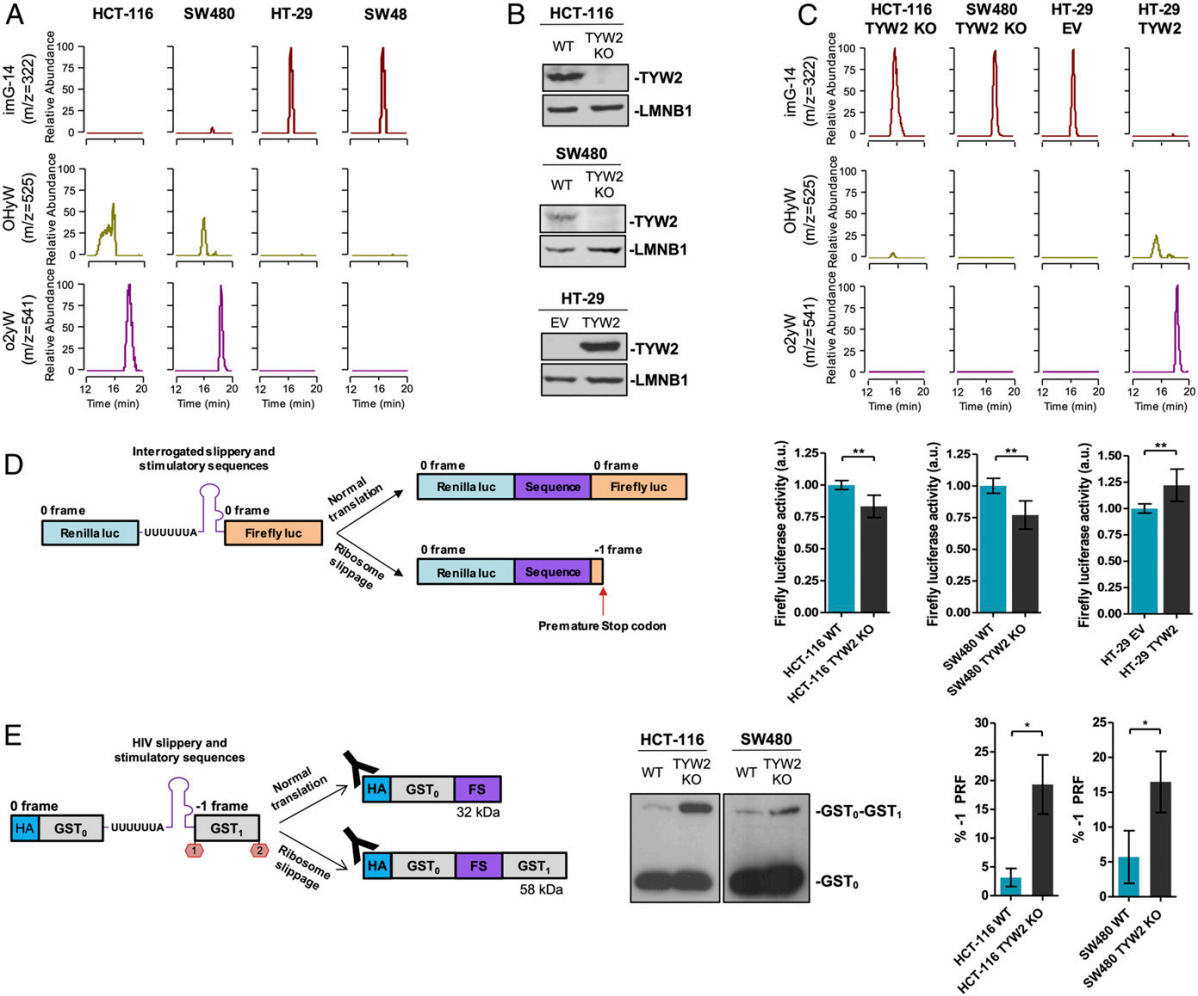
TYW2 epigenetic silencing in cancer cells mediates loss of hypermodified guanosine in tRNA and impairs ribosomal reading frame maintenance. (*A*) Nucleoside analysis of tRNAs by LC/MS showing that *TYW2* promoter hypermethylation and transcriptional inactivation in SW48 and HT-29 cell lines are associated with complete depletion of the fully modified wybutosine derivatives OHyW and o2yW and induce an accumulation of TYW2 substrate imG-14. *TYW2* unmethylated cell lines HCT-116 and SW480 present the fully modified residues and absence of the hypomodified intermediary imG-14. (*B*) Western blots showing efficient depletion of TYW2 expression in the unmethylated colon cell lines HCT-116 and SW480 on CRISPR/Cas9-mediated deletion (*Top*) and transfection-mediated recovery of TYW2 in the hypermethylated cell line HT-29 (Bottom). (*C*) LC/MS showing that CRISPR/Cas9 deletion of *TYW2* in HCT-116 and SW480 cells provokes a complete depletion of the fully modified wybutosine (yW) derivatives OHyW and o2yW and accumulation of the TYW2 substrate imG-14. The opposite scenario is observed on transfection-mediated recovery of TYW2 expression in HT-29 cells. WT, wild-type cells; EV, empty vector transfected cells. (*D*) In vitro assay to measure PRF at an mRNA containing a slippery sequence. (*Left*) Schematic representation of the dual luciferase reporter used to monitor −1 PRF events in the colon cell line models in which *Renilla* and firefly luciferases are separated by the slippery sequence from HIV and if ribosome slippage occurs, the reading frame changes and induces a premature stop codon detected by diminished firefly activity. (*Right*) CRISPR/Cas9-mediated loss of *TYW2* in HCT-116 and SW480 cells caused a significant reduction in firefly luciferase activity, whereas transfection-mediated recovery of TYW2 in the hypermethylated/silenced cell line HT-29 increased firefly luciferase activity. Firefly luciferase activity was compared using an unpaired two-tailed Student’s *t* test. **P < 0.01. (*E*, *Left*) Schematic representation of the dual-GST reporter used to evaluate −1 PRF events in colon cancer cell lines on CRISPR/Cas9-mediated *TYW2* depletion. Two GST coding sequences are separated by the slippery sequence from HIV, and the second GST is translated only if ribosome slippage occurs. (*E*, *Middle*) Representative Western blot of the reporter construct using anti-HA antibody in HCT-116 and SW480 cell line models. Western blots were performed in triplicate. (*E*, *Right*) Quantification of three independent replicates to evaluate the percentage of −1 PRF in each cell line model. The data represent the fraction between the intensity of the dual GST protein band (upper band) against the sum of intensities of the dual GST protein and single GST protein (lower band). Statistical differences among proportions were calculated using an unpaired two-tailed Student’s *t* test. *P < 0.05.

After detecting the presence of *TYW2* CpG island hypermethylation-associated transcriptional loss in colorectal cancer cell lines, we studied its contribution to the chemical modification status of G37 using tRNA-associated liquid chromatography-mass spectrometry (LC/MS) (26). We first assessed the profile of the modified nucleosides of G37 according to the *TYW2* CpG island methylation status in the studied colorectal cancer cell lines. We observed that the fully hypermodified residues OHyW and o2yW were present in the *TYW2* unmethylated/expressing cells HCT-116 and SW480 (Fig. 2*A*), whereas the intermediate modified nucleoside imG-14, the direct substrate of TYW2 (Fig. 1*A*), was absent (Fig. 2*A*). The opposite scenario was observed in the *TYW2* hypermethylated/silenced cell lines HT-29 and SW48, which showed an accumulation of the TYW2 substrate imG-14 and a loss of the hypermodified OHyW and o2yW (Fig. 2*A*).

To further interrogate the association between TYW2 expression and the chemical modification status of G37, we generated two stable *TYW2* knockout models in the unmethylated cell lines HCT-116 and SW480 to mimic the effects of the observed transcriptional silencing. In this approach, we used the CRISPR/Cas9 system to generate a deletion within *TYW2* gene body and confirmed the removal of a 234-bp fragment by Sanger sequencing (*SI Appendix*, Fig. S3*A*) and by genomic PCR (*SI Appendix*, Fig. S3*B*). The effectiveness of the knockout was confirmed by Western blot analysis, which showed no TYW2 protein expression in HCT-116 and SW480 *TYW2* KO cell lines (Fig. 2*B*). On CRISPR/Cas9-mediated loss of *TYW2* in HCT-116 and SW480, we found an accumulation of the TYW2 substrate imG-14 and loss of hypermodified OHyW and o2yW (Fig. 2*C*), mimicking the effects of the DNA methylation-associated transcriptional silencing in the HT-29 and SW48 cell lines (Fig. 2*A*). We also performed the reverse experiment in which we restored expression of TYW2 in the hypermethylated/silenced cell line HT-29 by stable transfection (Fig. 2*B*). The LC/MS approach resulted in the opposite scenario as seen in the CRISPR/Cas9 deletion model: the recovery of TYW2 expression by transfection depleted the intermediate imG-14 nucleoside and caused the appearance of OHyW and o2yW hypermodified residues (Fig. 2*C*), mimicking the pattern observed in the *TYW2* unmethylated/expressing cell lines HCT-116 and SW480 (Fig. 2*A*). The modified nucleoside yW-86 was undetectable in all the samples studied (*SI Appendix*, Fig. S4), supporting the reported kinetics of the pathway that rapidly transforms this residue to the hypermodified OHyW and o2yW nucleosides (21).

Hypermodified guanosine at position 37 of tRNA, such as wybutosine in yeast, is known to help maintain the reading frame during translation by preventing −1 PRF events at mRNAs that contain a slippery sequence (12). Thus, we wondered whether the appearance of hypomodified yW on TYW2 epigenetic inactivation could affect this mechanism, which preserves the proper mRNA-to-protein translation. To investigate this phenomenon in our human cancer cells, we used a dual luciferase reporter system as described previously (27). In this model, *Renilla* and firefly luciferases are separated by the widely known slippery sequence from HIV (28), and if ribosome slippage occurs, changing the reading frame and inducing a premature stop codon, the firefly activity is abolished (Fig. 2*D*). Using this reporter to monitor the reading frame maintenance of the described sequence, we found that CRISPR/Cas9-mediated loss of *TYW2* in HCT-116 and SW480 cells provoked a significant reduction in firefly luciferase activity (Fig. 2*D*). In the opposite experiment, transfection-mediated restoration of TYW2 in the hypermethylated/silenced cell line HT-29 increased firefly luciferase activity compared with empty vector-transfected cells (Fig. 2*D*).

We also used a second programmed frameshift reporter based-assay to evaluate the production of proteins resulting from −1 PRF events (12). In this dual glutathione S-transferase (GST) construct, two GST coding sequences are separated by the HIV slippery sequence and an in-frame stop codon before the second GST (Fig. 2*E*). In the event of ribosome slippage and alteration of the reading frame, this stop codon is ignored, and the second GST is translated, generating a larger protein. These proteins can be detected by Western blot analysis, and frameshifting frequency can be inferred by calculating the percentage of the large protein production (frameshift product) over the total protein production (the sum of the dual GST/large protein and the single GST/short protein) (Fig. 2*E*). Using this reporter, we observed a significant increase in the frameshifting frequency of the *TYW2* CRISPR/Cas9 KO HCT-116 and SW480 cells compared with the *TYW2* unmethylated/expressing cells (Fig. 2*E*). Thus, losing TYW2 and hypermodified yW in colon cancer cells confers a phenotype prone to ribosomal frameshifting events.

### Transcriptomic Characterization of RNA Slippery Sequences and Ribosome Frameshift on TYW2 Epigenetic Loss in Cancer Cells.

There is a balance between mRNA production and degradation that confers cellular homeostasis and adaptation. Among the different mechanisms involved in this equilibrium, PRF may serve as an mRNA abundance control mechanism by inducing a premature stop codon and mRNA degradation via NMD (16). The physiological status quo for mRNA levels is distorted in human tumors, and we wondered whether TYW2 epigenetic loss by generation of hypomodified guanosine and −1 PRF could induce aberrant mRNA degradation in colon cancer.

**Fig. 3.**
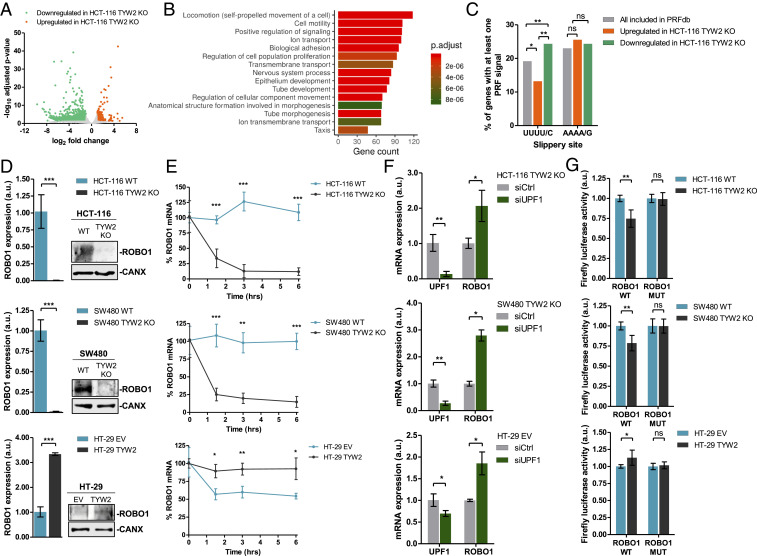
Transcriptomic identification of transcripts undergoing degradation on ribosome frameshift-mediated RNA decay and characterization of the ROBO1 candidate. (*A*) Volcano plot summarizing the results of the RNA-seq experiment to find differentially expressed transcripts in *TYW2* CRISPR/Cas9-depleted HCT-116 cells compared with wild-type HCT-116 cells. (*B*) GO analysis of Biological Process categories in the transcripts down-regulated on *TYW2* depletion in HCT-116 cells shows enrichment of GO Biological Process categories related to cell migration, including “locomotion,” “cell motility,” and “biological adhesion.” (*C*) Down-regulated transcripts in *TYW2* KO cells are enriched in −1 PRF sites containing at least one UUUU/C slippery sequences that are present in tRNA^Phe^ (*Left*); however, no enrichment is observed for transcripts containing AAAA/G slippery sequences, the codon for tRNA^Lys^ (*Right*). Statistical differences among proportions were calculated using Fisher’s exact test. ns, not significant; *P < 0.05. (*D*) ROBO1 mRNA and protein levels in the studied experimental models. (*Left*) *TYW2* CRISPR/Cas9 depletion in HCT-116 and SW480 cells down-regulated *ROBO1* mRNA as determined by qRT-PCR. (*Right*) Western blot assay showing the ultimate loss of ROBO1 protein in the *TYW2* KO cells. In the opposite model, TYW2 transfection-mediated recovery in HT-29 cells raised ROBO1 transcript and protein levels. For all cases, the qRT-PCR data shown represent the mean ± SD of at least three biological replicates analyzed using an unpaired two-tailed Student’s *t* test. ***P < 0.001. (*E*) RNA stability determination by the actinomycin D chase assay. A reduction in *ROBO1* transcripts levels is observed on CRISPR/Cas9-mediated deletion of *TYW2* HCT-116 and SW480 cells, but not in wild-type cells. In the reverse experiment, TYW2 transfection-mediated restoration in HT-29 cells stabilized *ROBO1* transcript levels. For all cases, data shown represent the mean ± SD of at least four biological replicates analyzed using the unpaired two-tailed Student’s *t* test at each time point. (*F*) Nonsense-mediated mRNA decay inhibition using a siRNA against *UPF1* in *TYW2* CRISPR/Cas9-depleted HCT-116 and SW480 cells and in empty vector (EV) transfected HT-29 cells resulted in *ROBO1* mRNA up-regulation as assessed by qRT-PCR. Data shown represents the mean ± SD of biological triplicates analyzed by unpaired two-tailed Student’s *t* test. *P < 0.05; **P < 0.01. (*G*) In vitro assay to measure PRF events at the cloned *ROBO1* mRNA containing the slippery and stimulatory sequences in the generated dual luciferase vector. HCT-116 and SW480 cells harboring CRISPR/Cas9 deletion of *TYW2* showed a reduction in firefly luciferase activity compared with wild-type cells. In the reverse model, TYW2 transfection-mediated recovery in HT-29 cells increased firefly activity. No differences in firefly activity between conditions were observed when phenylalanine codons of the slippery heptamer were mutated to leucine. ROBO1 WT, wild-type phenylalanine codons; ROBO1 MUT, mutant-introduced leucine codons. *P* values correspond to two-tailed unpaired Student’s *t* test. ns, not significant; *P < 0.05; **P < 0.01.

To explore this, we performed RNA-seq in the *TYW2* unmethylated/expressing HCT-116 cells and its derived cell line harboring CRISPR/Cas9-mediated loss of *TYW2*. We observed that the experimentally induced depletion of *TYW2* in HCT-116 cells altered the levels of 2,370 transcripts, mostly inducing transcript down-regulation (in 86%; 2,046 of 2,370) (Fig. 3*A*). Gene set enrichment analysis (GSEA) using Gene Ontology (GO) signature collections in this set of down-regulated transcripts demonstrated an overrepresentation of GO biological processes related to cell migration (e.g., “locomotion,” “cell motility,” “biological adhesion”) (Fig. 3*B*). The transcripts down-regulated on *TYW2* CRISPR/Cas9 deletion were also commonly down-regulated in those colorectal cell lines in which the TYW2 enzyme was naturally silenced by promoter CpG island hypermethylation (*SI Appendix*, Fig. S5). Among the down-regulated transcripts, 109 (*SI Appendix*, Table S1) were included in a database of computationally predicted eukaryotic programmed −1 PRF signals (PRFdb) (29) and contained at least one predicted slippery sequence with UUUU/C, the codon decoded by tRNA^Phe^ and thus regulated by the hypermodification of G37. Importantly, these transcripts with the −1 PRF signal and the UUUU/C sequence were significantly enriched among all down-regulated transcripts compared with up-regulated transcripts and all the genes included in the PRFdb database (Fig. 3*C*). Serving as an important negative control, the transcripts with slippery sequences containing AAAA/G, the codon for another frameshift-related tRNA (tRNA^Lys^), were not enriched in the transcripts down-regulated on TYW2 loss (Fig. 3*C*). In a similar manner, and as an additional negative control, transcripts that did not contain the UUU or UUC codons were not enriched in this down-regulated group (*SI Appendix*, Fig. S6). Interestingly, the proteins encoded by the down-regulated transcripts with the −1 PRF signal frequently interacted with proteins derived from the down-regulated transcripts without the motif (*SI Appendix*, Fig. S7), an event potentially affecting RNA fate. Thus, the 109 transcripts identified constitute bona fide candidates for direct targeting by TYW2 epigenetic loss in the colon cancer cells.

Among the identified TYW2 targets, we selected the roundabout guidance receptor 1 (ROBO1) for further validation and study. ROBO1 was selected because it is reportedly lost in some colorectal tumors (30, 31) and because of its proposed role as an inhibitor of cell migration and epithelial-to-mesenchymal transition (EMT) (32, 33). We first validated the RNA expression levels observed in the RNA-seq approach by quantitative real-time RT-PCR, and found that the *TYW2* unmethylated/expressing HCT-116 and SW480 cells expressed the *ROBO1* transcript, whereas CRISPR/Cas9-mediated inactivation of *TYW2* induced the loss of *ROBO1* transcript levels (Fig. 3*D*). This finding was also mirrored at the protein level by Western blot analysis showing that ROBO1 protein was present in the wild-type HCT-116 and SW480 cells, whereas it was lost on experimental depletion of *TYW2* (Fig. 3*D*). The reverse profile of ROBO1 was observed in *TYW2* hypermethylated HT-29 cells; the recovery of TYW2 expression by transfection raised the RNA and protein levels of ROBO1 compared with cells transfected with the empty vector (Fig. 3*D*). Remarkably, using actinomycin D (Fig. 3*E*) and alpha-amanitin (*SI Appendix*, Fig. S8) chase assays, we demonstrated a loss of *ROBO1* transcript stability on *TYW2* CRISPR/Cas9 deletion in HCT-116 and SW480 cells (Fig. 3*E* and *SI Appendix*, Fig. S8), but increased ROBO1 transcript stability on TYW2 transfection in HT-29 cells (Fig. 3*E* and *SI Appendix*, Fig. S8), thus supporting the role of TYW2 in the regulation of *ROBO1* mRNA.

We considered that the reduced stability of *ROBO1* mRNA might be associated with NMD (34, 35) induced by the ribosome frameshifting originating at a premature stop codon located 52 nt upstream of the subsequent exon-exon junction. To test this idea, we used siRNA targeting *UPF1* to deplete the RNA helicase responsible for initiating NMD (34, 35). We observed that the inhibition of NMD activity in all the cell line models with TYW2 loss (i.e., the CRISPR/Cas9-depleted HCT-116 and SW480 cells and the hypermethylated HT-29 cells) resulted in up-regulation of *ROBO1* levels (Fig. 3*F*), indicating that NMD contributes to the loss of ROBO1 in TYW2-deficient cells. As endogenous positive controls for the assay, we used transcripts reportedly targeted by NMD inhibition (36) (*SI Appendix*, Fig. S9). As an internal control, we did not observe any changes in RNA stability and NMD assays for transcripts that did not change on TYW2 loss in the RNA-seq analyses (*SI Appendix*, Fig. S10 *A*–*C*). Finally, we further assessed the impact of TYW2 loss in the generation of ROBO1 frameshifting events by cloning the predicted slippery and stimulatory sequences of ROBO1 in the produced dual luciferase vector (Fig. 2*D*), as described in *SI Appendix*, Fig. S11. We observed that the CRISPR/Cas9-mediated loss of TYW2 caused decreased firefly luciferase activity for ROBO1 compared with the wild-type cells (Fig. 3*G*). In the opposite model, TYW2 restoration by transfection in the hypermethylated HT-29 cells increased firefly activity for ROBO1 (Fig. 3*G*). Importantly, and to ensure that differences in reading frame maintenance between conditions were due to specific slippage of tRNA^Phe^, we also generated a luciferase reporter for ROBO1 harboring a mutation in the phenylalanine codons in the slippery heptamer (*SI Appendix*, Fig. S11). This mutation abolished the effect of *TYW2* CRISPR/Cas9 deletion (HCT-116 and SW480 KO cells) or TYW2 epigenetic silencing (HT-29 hypermethylated cells) on firefly activity (Fig. 3*G*), similar to the action observed in the TYW2-expressing cells such as the unmethylated wild-type HCT-116 and SW80 cells or the methylated HT-29 cells in which TYW2 expression was restored by transfection (Fig. 3*G*). Thus, the loss of the multifaceted cancer gene ROBO1 on TYW2 epigenetic inactivation provides an illustrative example of how alterations of tRNA chemical modifications might contribute to tumorigenesis.

### TYW2 Epigenetic Inactivation Highlights Early-Stage Primary Colorectal Tumors with Poor Clinical Outcome.

**Fig. 4.**
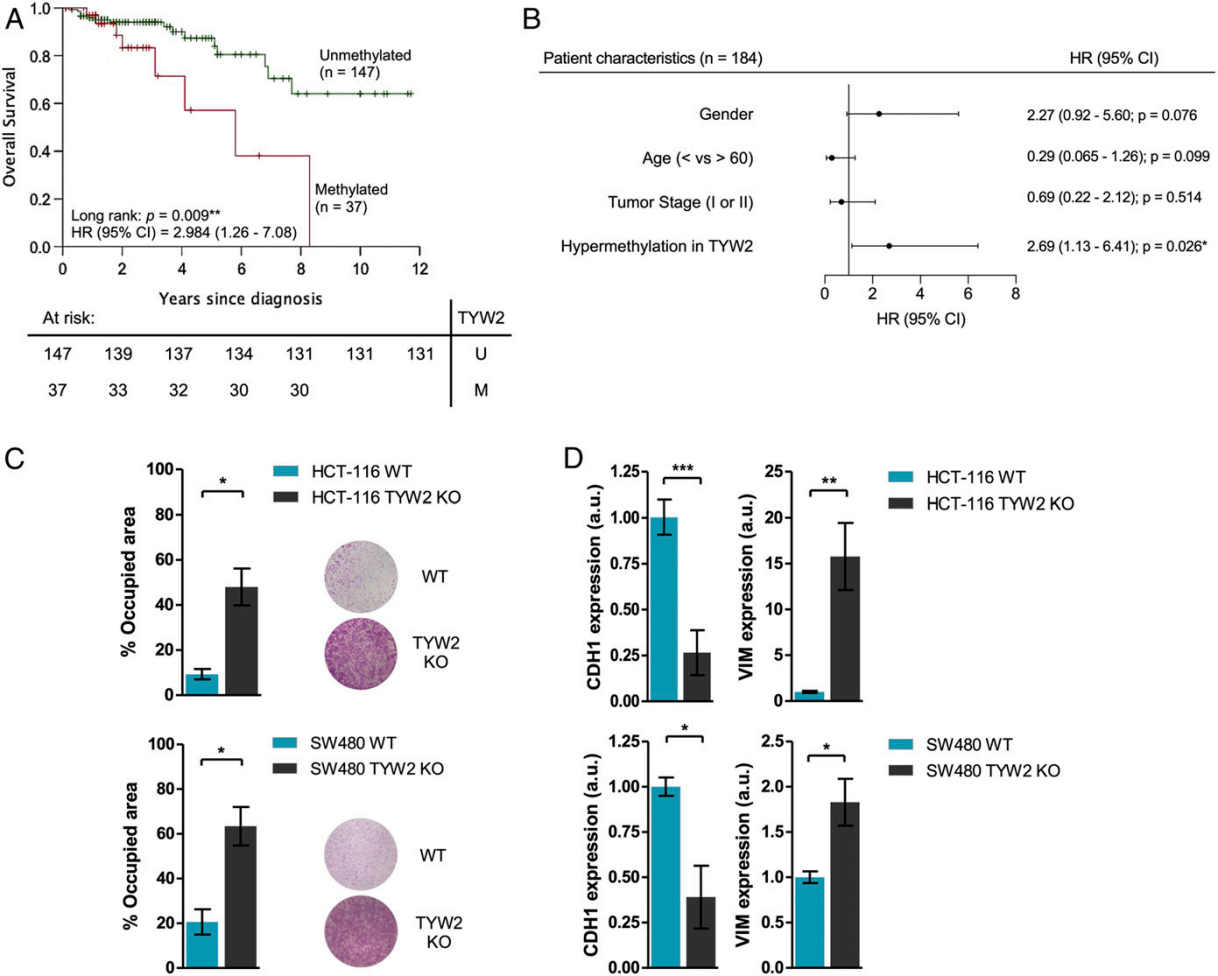
*TYW2* epigenetic loss is associated with early-stage primary colorectal tumors with poor clinical outcome. (*A*) Kaplan–Meier curve showing that the presence of *TYW2* hypermethylation in patients with early-stage colorectal cancer (*n* = 184) was significantly associated with shorter overall survival (*P* = 0.009). Green line, unmethylated cases; red line, methylated cases. **P < 0.01. (*B*) Forest plot representation of the Cox proportional hazard regression models demonstrating that TYW2 hypermethylation is an independent prognostic factor of poor overall survival in patients with early-stage colorectal cancer (HR = 2.69; 95% CI = 1.13 to 6.41; *P* = 0.026). ns, not significant; *P < 0.05; (*C*) Transwell assay to determine cell migration capability in the studied models. HCT-116 and SW480 cells carrying the CRISPR/Cas9 deletion of *TYW2* showed a significant increase in the number of migrated cells after 48 h compared with wild-type cells. Representative images of the Transwell membrane staining are shown. *P* values are those associated with an unpaired two-tailed Student’s *t* test. *P < 0.05. (*D*) Assessment of EMT features in the studied models using qRT-PCR levels of the E-cadherin (epithelial) and vimentin (mesenchymal) markers. The KOs of *TYW2* in the HCT-116 and SW480 cell lines showed a combined decrease in *E-cadherin* expression and enhancement *Vimentin* levels compared with wild-type cells, a transcript profile suggestive of the acquisition of EMT features. Data shown represent the mean ± SD of at least three biological replicates and were analyzed using an unpaired two-tailed Student’s *t* test. *P < 0.05; **P < 0.01; ***P < 0.001.

Having demonstrated the presence of *TYW2* promoter CpG island hypermethylation-associated transcriptional silencing and its role in the generation of hypomodified guanosine in tRNA^Phe^ and ribosome frameshift-associated RNA degradation, we studied if TYW2 epigenetic loss in primary colorectal carcinoma had any impact on outcome in these patients. To do so, we data-mined the colorectal carcinoma cases from the TCGA effort in which we had previously identified the presence of *TYW2* hypermethylation (Fig. 1*B*) and associated silencing (Fig. 1*C*) for the 371 available tumors with complete clinical information. For the entire population of colorectal tumors, *TYW2* promoter hypermethylation showed a trend toward an association with poor overall survival, but this was not statistically significant (*P* = 0.460, log-rank test) (*SI Appendix*, Fig. S12). However, when subdivided by stages, colorectal cancer patients with early-stage disease (*n* = 184, stage I and II) harboring TYW2 epigenetic inactivation showed significantly reduced overall survival (hazard ratio [HR] = 2.984; 95% confidence interval [CI] = 1.26 to 7.08; *P* = 0.009, log-rank test) (Fig. 4*A*). Most importantly, multivariate Cox regression analysis identified *TYW2* hypermethylation as an independent predictor of shorter overall survival in early-stage colorectal cancer (HR = 2.69; 95% CI = 1.13 to 6.41; *P* = 0.026) (Fig. 4*B*) compared with other patient characteristics that also have been associated with the clinical outcome (Fig. 4*B*).

Reinforcing these data, similar results were obtained when we interrogated *TYW2* expression levels in the same TCGA colorectal cohort. For the entire population of colorectal cancer patients, low *TYW2* expression levels showed a trend toward its association with poor overall survival, but this was not statistically significant (*P* = 0.320, log-rank test) (*SI Appendix*, Fig. S13*A*). However, when subdivided by stage, early-stage colorectal cancer patients with TYW2 low expression levels showed significantly reduced overall survival (HR = 4.78; 95% CI = 2.03 to 11.27; *P* < 0.001, log-rank test) (*SI Appendix*, Fig. S13*B*). As was shown for *TYW2* DNA methylation (Fig. 4*B*), multivariate Cox regression analysis identified low *TYW2* expression as an independent predictor of shorter overall survival in early-stage colorectal cancer (HR = 4.09; 95% CI = 1.71 to 9.79; *P* = 0.002) compared with other patient characteristics that also have been associated with clinical outcome (*SI Appendix*, Fig. S13*C*).

These results could suggest that *TYW2* hypermethylation in patients with early-stage colorectal cancer might pinpoint those tumors that, even at this early stage, contain transformed cells that are more prone to escape from the primary site and disseminate the disease. To check this, we went back to our colorectal cancer cell line models to assess their cellular migration capacities. Using a Transwell assay, we found that CRISPR/cas9-mediated deletion of *TYW2* in the unmethylated and expressing HCT-116 and SW480 colon cancer cell lines induced a significant increase in the migration potential of the obtained TYW2-deficient cells (Fig. 4*C*). Most importantly, we wondered whether this type of cellular reprogramming was occurring on TYW2 loss in epithelial tumors such as colon cancer, in which the migration, cellular detachment, and invasion capacities involve the EMT (37, 38).

Most molecular pathways controlling the EMT converge in E-cadherin, a cell adhesion protein crucial for maintaining epithelial structure (37, 38), whereas expression of vimentin is a hallmark of mesenchymal features (37, 38). In this regard, we found that *TYW2* CRISPR/Cas9-mediated deletion in both HCT-116 and SW480 colon cancer cells induced down-regulation of E-cadherin and up-regulation of vimentin (Fig. 4*D*). Interestingly, and in line with the role of TYW2 loss in ROBO1 down-regulation (Fig. 3 *D*–*G*) and in the proposed EMT inhibitory activity of this protein (32, 33), restoration of ROBO1 expression by transfection in the *TYW2* CRISPR/Cas9-deleted HCT-116 and SW480 cells rescued the phenotype, inducing the opposite effect with E-cadherin up-regulation and vimentin down-regulation (*SI Appendix*, Fig. S14 *A* and *B*). CRISPR/cas9-mediated deletion of TYW2 did not change the cell cycle, as assessed by BrdU and 7AAD incorporation, p21 expression as a marker of cell arrest, or apoptosis measured by PARP cleavage (*SI Appendix*, Fig. S15 *A*–*C*). Thus, TYW2 loss is associated with increased migration capacity and the emergence of EMT features, both of which have central roles in metastasis development, the leading cause of cancer-associated mortality. These observations are in line with the identified poor clinical outcomes of colorectal cancer patients with TYW2 hypermethylation-associated inactivation.

## Discussion

tRNAs are extremely critical biological molecules that, working together with the ribosomes, allow translation of the genetic code into amino acids. In humans, there are 47 different types of tRNAs for 20 standard amino acids, in addition to selenocysteine (39). The amount of each tRNA is tightly controlled to match the codon usage of mRNAs to optimize protein expression according to the cellular necessities. For many years, it was thought that tRNAs were passive bystanders in cellular transformation, and that changes in tRNA levels were simply a consequence of the high proliferative status of tumoral cells. However, an increasing amount of new data show that tRNA might play an active role in tumorigenesis (40, 41). These results include, among others, the presence of mitochondrial tRNA mutations in cancer cells (42), the antiapoptotic activity of tRNAs (43), and the detection of tRNA derivatives in transformed cells that exert oncogenic or tumor-suppressor functions (44, 45).

Another important twist involving tRNAs in cancer origin and development is the recognition that an aberrant pattern of chemical modifications in the tRNA molecule occurs in neoplasia, causing altered protein translation (1, 10). These findings can now be categorized within the emerging field of epitranscriptomics, defined as the chemical modifications that affect RNA activity. This area is booming because RNA modifications seem to play an essential role in cellular differentiation (46) as well as in tumorigenesis (1, 47). Human RNAs are modified in many ways (e.g., 5-methylcytosine, N6-methyladenosine, N1-methyladenosine, pseudouridine, etc.) (48), and disruptions of the proteins involved in adding, removing, or binding to these chemical marks are being identified in human cancers (49), such as the ribosomal RNA modifiers DKC1 (50) and NSUN5 (51). In this regard, tRNA itself constitutes one of the most heavily modified molecules, including some highly specific nucleotides (6). Examples of tRNA modifiers involved in carcinogenesis include ELP3, which is up-regulated in different tumor types and acts on the Wnt pathway (52) and also has been related to metastasis formation (53); the tRNA methyltransferase 9B, which has been proposed to exert a role in tumor suppression (54); and the tRNA methyltransferase NSUN2, which undergoes gene amplification-associated overexpression in human tumors (55). Interestingly, modifications within the anticodon stem-loop at position 37 of tRNA is crucial for a correct base pairing interaction (56), and deficient hypermodification of G37 in tRNA^Phe^, without any evident cause, was reported in cancer models decades ago (17–19). Here we demonstrate that the generation of hypomodified G37 in tRNA^Phe^ is due to the cancer-specific epigenetic inactivation of the tRNA- modifying enzyme TYW2.

Promoter CpG island hypermethylation-associated silencing of *TYW2* was observed across a wide spectrum of human cancer types, with colorectal tumors the most common type of tumor in which this epigenetic defect was identified. Most importantly, the loss of TYW2 was associated not only with the lack of hypermodified forms of G37 (e.g., OHyW, o2yW), but also with increased ribosome frameshift, leading to transcript degradation. The transcriptomic assay showed that numerous RNAs were targeted by this tumor-specific event induced on TYW2 silencing. The tumor suppressor ROBO1 is a robust example of key genes that can be hampered by this process. Even more important from the clinical side, the DNA methylation-linked loss of TYW2 was associated with poor clinical outcome in colorectal cancer patients at early stages of the disease. Thus, TYW2 could be proposed as a biomarker of early dissemination for those tumors that at first glance seem confined to the primary site but in reality might have escaped from its original niche. These results could be linked to the observed appearance of higher migration rates and mesenchymal features in those cancer cells harboring TYW2 epigenetic silencing. Given the emergence of many epigenetic drugs that restore the activity of genes inactivated by DNA hypermethylation (24), it is also tempting to speculate that the use of inhibitors of DNA methylation would rescue TYW2 deficiency, restoring ribosome and RNA base pairing, and avoid the migration-prone features of these cells, thereby reducing the mortality of the identified high-risk patients that present with an otherwise apparently localized disease.

## Materials and Methods

The materials and methods used in this study are summarized here; more detailed information is available in *SI Appendix*, *Materials and Methods*. The sequences of all primers, as well as references for all antibodies, reagents and commercial assays, are provided in *SI Appendix*, Table S2.

### DNA Methylation Analyses.

Gene promoter DNA methylation status was determined by DNA methylation microarrays and bisulfite genomic sequencing.

### Expression Analyses.

RNA expression was assessed by qRT-PCR, and protein expression was determined by Western blot analysis.

### TYW2 Cellular Models.

In unmethylated HCT-116 and SW480 cell lines, TYW2 expression was abolished using the CRISPR/Cas9 system as described previously (57). For stable TYW2 restoration in the hypermethylated HT-29 cell line, the TYW2 cDNA sequence was cloned into pLVX-IRES-ZsGreen plasmid (Clontech) and delivered using lentivirus containing this construct.

### tRNA Nucleosides LC-MS.

tRNA from cellular models were extracted and digested, and nucleosides were analyzed by liquid chromatography-mass spectrometry as described previously (26).

### −1 PRF Evaluation.

−1 PRF events were evaluated using a dual luciferase reporter as described previously (27). In brief, *Renilla* and firefly luciferases were cloned into the pcDNA4 T/O vector, separated by the slippery sequence of interest. In the event of a −1 PRF event, the new reading frame will generate a premature stop codon, and firefly activity will be abolished.

### RNA-Seq.

Total RNA from wild-type and CRISPR/Cas9-mediated *TYW2* KO HCT-116 cells was used for RNA-seq analyses. Genes were considered to be differentially expressed when the log2 fold change was <−1.0 or >1.0 and the adjusted *P* value was < 0.05. GO biological processes gene sets included in the GSEA signature database were used to perform an overrepresentation analysis on the down-regulated transcripts. Coding genes were queried to the PRFdb (29) and interrogated for the presence of putative slippery sequences.

### Migration Assays.

Cell migration capacity was assessed by a Transwell assay.

### Cell Cycle and Apoptosis Assays.

The cell cycle was assessed by measuring BrdU incorporation and 7AAD staining, by immunoblotting of p21 and PARP cleavage.

### Statistics.

Spearman’s correlation, unpaired Student’s *t* test, or Fisher’s exact test were used as appropriate. Kaplan–Meier plots and the log-rank (Mantel–Cox) test were used to estimate overall survival. *P* < 0.05 was considered to indicate statistical significance.

## Supplementary Material

Supplementary File

Supplementary File

Supplementary File

Supplementary File

## Data Availability

All study data are included in the main text and *SI Appendix*.
