# Supplementary material for: Epigenetic loss of the transfer RNA-modifying enzyme TYW2 induces ribosome frameshifts in colon cancer
Source: Proc Natl Acad Sci U S A. 2020 Aug 10;117(34):20785–93. doi: 10.1073/pnas.2003358117 (PMC7456070; doi:10.1073/pnas.2003358117)
Supplement: Supplementary File [file pnas.2003358117.sapp.pdf]

SUPPORTING INFORMATION FOR:

Epigenetic Loss of the tRNA-Modifying Enzyme TYW2 Induces Ribosome Frameshifts in Colon Cancer

Margalida Rosselló-Tortella[a,b], Pere Llinàs-Arias[a], Yuriko Sakaguchi[c], Kenjyo Miyauchi[c], Veronica Davalos[a], Fernando Setien[a], Maria E. Calleja-Cervantes[a], David Piñeyro[a,d], Jesús Martínez-Gómez[a], Sonia Guil[a,b], Ricky Joshi[a], Alberto Villanueva[e], Tsutomu Suzuki[c] and Manel Esteller[a,d,f,g,1]

[a]Cancer Epigenetics Group, Josep Carreras Leukaemia Research Institute (IJC), 08916, Badalona, Barcelona, Catalonia, Spain

[b]Germans Trias i Pujol Health Science Research Institute (IGTP), 08916 Badalona, Barcelona, Catalonia, Spain

[c]Department of Chemistry and Biotechnology, Graduate School of Engineering, University of Tokyo, Bunkyo-ku, 113-8656 Tokyo, Japan

[d]Centro de Investigacion Biomedica en Red Cancer (CIBERONC), 28029 Madrid, Spain

[e]Translational Research Laboratory, Catalan Institute of Oncology (ICO), Bellvitge Biomedical Research Institute (IDIBELL), 08908 L'Hospitalet, Barcelona, Catalonia, Spain

[f]Institucio Catalana de Recerca i Estudis Avançats (ICREA), 08010 Barcelona, Catalonia, Spain

[g]Physiological Sciences Department, School of Medicine and Health Sciences, University of Barcelona (UB), 08907 Barcelona, Catalonia, Spain

[1]Corresponding author:

Manel Esteller

Email: mesteller@carrerasresearch.org

This PDF file includes:

- Supplementary materials and methods
- Supplementary references
- Figures S1 to S15
- Tables S1 and S2

Other supplementary materials for this manuscript include the following:

Datasets S1 to S3

www.pnas.org/cgi/doi/10.1073/pnas.2003358117

Supplementary Materials and Methods

Cell lines

Colon cancer cell lines (SW48, HT-29, HCT-116 and SW480) were purchased from the American Type Culture Collection (ATCC). All of them were cultured in Dulbecco's Modified Eagle's Medium (DMEM) supplemented with 10% fetal bovine serum (FBS) and 1% penicillin/streptomycin at 37°C and 5% CO_2. All cell lines were authenticated by short tandem repeat profiling (LGS Standards SLU) and tested for the absence of mycoplasma. For DNA demethylating treatment, SW48 and HT-29 cells were cultured with 1 µM 5-aza-2'-deoxycytidine (Sigma) for 72 hours. For mRNA decay analyses, cells were incubated with actinomycin D (Sigma) at 10 µg/mL and collected at 0, 1.5, 3 and 6 hours after treatment release or with alpha-amanitin (MedChemExpress) at 20 µg/mL and collected at 0, 4, 8 and 24 hours after treatment release.

DNA methylation analyses

DNA methylation status at gene promoter was determined by DNA methylation microarrays and bisulfite genomic sequencing. The DNA methylation microarray used was the Infinium HumanMethylation450 BeadChip (Illumina). In silico methylation analyses of TRMT5, TYW1, TYW2, TYW3, TYW4 and TYW5 genes in primary tumor samples from TCGA and cancer cell lines (1) were performed considering the CpG sites located at the 5'-end regulatory regions. DNA methylation values of individual CpG sites for TCGA cohorts of primary tumors and healthy tissues and cell lines are listed in **Datasets S1 to S3**. For bisulfite genomic sequencing, genomic DNA was converted using the EZ DNA Methylation-Gold Kit (ZYMO Research). Bisulfite PCR products were transformed into competent bacteria and a minimum of 10 clones were sequenced to calculate methylation frequency. Bisulfite PCR primers used are listed in **SI Appendix Table S2**. Results were analyzed with BioEdit software and methylated cytosines were mapped using BSMap software.

Expression analyses

RNA expression was assessed by quantitative reverse transcription PCR (qRT-PCR). Total RNA was extracted from cell pellets using the SimplyRNA kit (Promega) in the automated Maxwell RSC device (Promega). 2 µg of total RNA were converted to cDNA using the RevertAid First Strand cDNA synthesis kit (Thermofisher Scientific) according to manufacturers' specifications. Quantitative PCR was conducted using SYBR Green PCR Maser Mix (Applied Biosystems). All qRT-PCR were normalized using GAPDH expression as endogenous control except for alpha-amanitin mRNA decay, which was normalized using 28S rRNA expression. All qRT-PCR primers are listed in **SI Appendix Table S2**.

Protein expression was determined by western blotting. Total protein was extracted with Laemli sample buffer (62.5 mM Tris-HCl pH 6.8, 25% glycerol, 2% SDS, 0.01% bromophenol blue, 5% β-mercaptoethanol) or RIPA buffer (20 mM Tris-HCl pH 7.5, 150 mM NaCl, 1 mM Na_2EDTA, 1 mM EGTA, 1% NP-40), and Lamin B1 (LMNB1), β-actin (ACTB) or Vinculin (VCL) were used as loading controls. For membrane fraction enrichment to perform ROBO1 western blot, fresh cell pellets were fractioned using ProteoExtract® Subcellular Proteome Extraction Kit (Merck) and Calnexin (CANX) was used as endogenous control. All primary antibodies used are listed in **SI Appendix Table S2**.

Gene expression modulation in cell lines

TYW2 expression was abolished in unmethylated cell lines using the CRISPR/Cas9 system as previously described (2). Two different sgRNA were cloned into pSpCas9(BB)-2A-GFP vector (Addgene) and transfected simultaneously in HCT-116 and SW480 cell lines using JetPrime® Transfection Reagent. Green positive cell lines were isolated by cell sorting to establish clonal cell lines after 48 hours of transfection and left for clonal expansion for approximately three weeks. Knockout clones were screened by amplification and sequencing of the sgRNA target region within TYW2 gene body. sgRNA constructions and primers used for amplification of the targeted region are listed in **SI Appendix Table S2**. For stable TYW2 restoration in the hypermethylated HT-29 cell line, TYW2 cDNA was obtained from the HCT-116 cell line and cloned into pLVX-IRES-ZsGreen1 plasmid (Clontech) with a Kozak sequence and a Flag-tag at the C-terminus of the protein. Lentivirus containing this construct were produced by cotransfecting HEK293T cells with recombinant pLVX-IRES-ZsGreen, psPAX2 (Addgene) and pMD2.G (Addgene) using JetPrime® Transfection Reagent. After 72 hours, virus-containing media was collected, filtered and delivered to HT-29 cells. Green positive infected cells were purified by cell sorting after five passages. TYW2 cloning primers are listed in **SI Appendix Table S2**.

For siRNA transfection, 200.000 cells were transfected with 50 nM siRNA against UPF1 (Thermofisher Scientific siRNA ID 12290) or a negative control using JetPrime® Transfection Reagent according to manufacturer's instructions. After 48 hours, cells were collected by cell scraping and RNA extraction was conducted as previously described.

For transient ROBO1 overexpression, ROBO1 cDNA from SW480 WT cell line was cloned into pcDNA4 T/O vector (Thermofisher Scientific) with a Kozak sequence and a HA-tag at the C-terminus of the protein. This construct and the empty pcDNA4 T/O were transfected using JetPrime® Transfection Reagent following the manufacturer's indications. Cells were collected after 72 hours and RNA and protein were extracted as previously described.

tRNA nucleosides liquid chromatography - mass spectrometry (LC/MS)

Total RNA was extracted using TRIzol reagent as per manufacturer's instructions. Total RNA was subjected to urea-PAGE separation to isolate tRNA fraction. Next, tRNA were digested with Nuclease T_1 (Wako Pure Chemical Industries), phosphodiesterase I (Worthington Biochemical Corporation) and bacterial alkaline phosphatase (Takara Bio). Nucleosides were analyzed by liquid chromatography-mass spectrometry (LC/MS) as described previously (3) using QExactive and U3000 liquid chromatography system (Thermofisher Scientific). Briefly, nucleosides were separated on zic-cHILIC column (3um, 100Å,150×2.1 mm, Merck), and then ionized and detected by ESI-QExactive (3).

-1 programmed ribosome frameshift evaluation

-1 programmed ribosome frameshifting events were evaluated using a dual luciferase reporter (4) and a dual GST reporter (5) as described previously. For the dual luciferase experiment, renilla and firefly luciferases were cloned into pcDNA4 T/O vector (Thermofisher Scientific) separated by the slippery sequence of interest. Should the ribosome retrocede, the new reading frame with generate a premature stop codon and firefly activity will be abolished. All primers used for luciferase amplification and generation of the interrogated slippery sequences are listed in the **SI Appendix Table S2**. This reporter was transfected into 10.000 cells plated in 96-well white plates after overnight adherence using JetPrime® Transfection Reagent according to manufacturer's instructions. Firefly and renilla luminescence were determined after 72 hours using the Dual Glo™ Luciferase Assay System (Promega) with the following change on the manufacturers' standard protocol: 45 µL of each reagent were used instead of the same volume of media. A minimum of four biological replicates with technical triplicates were analyzed.

For the dual GST reporter, two GST coding sequences in different reading frames were cloned into pcDNA4 T/O (Thermofisher Scientific), separated by the HIV slippery and stimulatory sequences and an in frame stop codon. The first GST was preceded by an HA-tag. All primers used for GST amplification and generation of the interrogated slippery sequences are listed in the **SI Appendix Table S2**. Translation stops at the first stop codon, generating a 28 kDa protein with a single GST. In case of ribosome frameshift, the alteration of the reading frame eliminates the first stop codon and the second GST is translated, generating a dual GST protein of 52 kDa. This reporter was transfected into cells at 70% confluence in 6-well plates using JetPrime® Transfection Reagent following the manufacturer's indications. 72 hours after transfections, cells were collected and total protein was extracted, as previously described and reporter expression was evaluated by western blotting using anti-HA antibody. Frameshift frequency was considered to be the fraction of dual GST protein compared to the total protein generated form the reporter.

RNA-sequencing experiment and data filtering

Total RNA from HCT-116 wild-type and TYW2 CRISPR/Cas9 knockout was extracted on a Maxwell RSC device. 5 µg of total RNA from three biological replicates from each sample were used for RNA-sequencing (RNA-seq) analyses. The RNA-seq libraries were prepared from total RNA with TruSeq®Stranded mRNA LT Sample Prep Kit (Illumina). Each library was sequenced using TruSeq SBS Kit v4-HS, in paired-end mode with a read length of 2x76+8+8bp. We obtained between 60 and 80 million paired-end reads in a fraction of a sequencing lane on HiSeq2500 (Illumina) following the manufacturer's protocol. Raw reads were quality assessed and preprocessed using FASTQC (v0.11.7) and Trimmomatic (v0.36) software. Differential expression analysis was performed using DESeq2 Bioconductor package (v1.18.1) (6), in R (v3.4.3). Gene annotations were extracted from GENECODE (v28). Genes were considered to be differentially expressed when log2 fold change < -1.0 or > 1.0 and adjusted *p*-value < 0.05.

GO-biological processes gene sets included in the GSEA signature database were used to perform an over-representation analysis on the downregulated transcripts. Top ten gene clusters resulting from a hypergeometric test with FDR adjusted *p*-value <0.05 were considered. Coding genes were queried to the PRFdb (7) and interrogated for the presence of putative slippery sequences. To search for human transcripts without phenylalanine codons in their coding sequence, we downloaded "Protein-coding transcript translation sequences" from Gencode v34 (8) and we selected proteins without Phe in all their annotated transcripts. From this list of proteins without Phe and our list of candidate genes, we found the intersection to discover which of our candidate genes do not have Phe in any of their isoforms. We searched for all known interactions occurring in our 2046 downregulated candidates set using STRINGsb R package (v2.0.0) to interrogate STRINGdb v11 (9). We used the package functionality to calculate PPI enrichment *p* values and to find the best subnetworks by using the "fastgreedy" clustering algorithm. Network plots were constructed using igraph R package (v.1.2.5).

Migration assays

Cell migration capacity was assessed by transwell assay. 100.000 HCT-116 cells and 200.000 SW480 cells were seeded in serum-free media in the upper chamber of an 8 µm pore transwell polycarbonate membrane insert and left for migration to the serum-containing lower chamber. Empty vector and ROBO1-transfected cells were collected and seeded in serum-free media 24 hours after the transfection. After 48 hours, transwell membranes were fixed with 10% trichloroacetic acid for 1 hour and stained with 0.057% SRB for 30 minutes. ImageJ was to calculate the percentage of membrane area occupied by cells to assess cell migration.

Cell cycle assays

Cell cycle was analyzed in Bromodeoxyuridine (BrdU) labelled cells using an APC anti-BrdU/7AAD staining using an APC BrdU Flow Kit (BD Biosciences, 552598). Briefly, cells were incubated with 10µM BrdU during 1h at 37ºC. Then, cells were washed, fixed and permeabilized following manufacturer's instructions. Samples were incubated with APC-labeled anti-BrdU antibody during 30 minutes at room temperature. Then total DNA was stained using 2uL of 7AAD solution. A minimum of 10,000 cells was analyzed per sample. Experiments were performed in triplicate.

Statistics

The association between variables were assessed by Spearman's correlation, unpaired Student's t-test or Chi-squared test as appropriate. Data normality was assessed using a Shapiro-Wilk test. Kaplan-Meier plots and Logrank (Mantel-Cox) tests were used to estimate overall survival (OS) through univariate and multivariate Cox proportional hazards regression models. Statistical analyses were carried out with R programming environment, GraphPad Prism 5 and IBM SPSS software. Values of $p < 0.05$ were considered statistically significant.

Data availability

HCT-116 wild-type and TYW2 CRISPR/Cas9-mediated knockout RNA-seq data have been deposited in the Sequence Read Archive repository (SRA) under project code PRJNA596997. Data can be accessed through the following web address: https://trace.ncbi.nlm.nih.gov/Traces/sra/sra.cgi

Supplementary references

1. Iorio F, et al. (2016) A Landscape of Pharmacogenomic Interactions in Cancer. *Cell* 166(3):740–754.
2. Ran FA, et al. (2013) Genome engineering using the CRISPR-Cas9 system. *Nat Protoc* 8(11):2281–2308.
3. Sakaguchi Y, Miyauchi K, Kang B Il, Suzuki T (2015) Nucleoside Analysis by Hydrophilic Interaction Liquid Chromatography Coupled with Mass Spectrometry. *Methods in Enzymology* (Elsevier Inc.), pp 19–28. 1st Ed.
4. Grentzmann G, Ingram JA, Kelly PJ, Gesteland RF, Atkins JF (1998) A dual-luciferase reporter system for studying recoding signals. *RNA* 4:479–486.
5. Carlson BA, et al. (1999) Transfer RNA modification status influences retroviral ribosomal frameshifting. *Virology* 255(1):2–8.
6. Love MI, Huber W, Anders S (2014) Moderated estimation of fold change and dispersion for RNA-seq data with DESeq2. *Genome Biol* 15(550). doi:10.1186/s13059-014-0550-8.
7. Belew AT, Hepler NL, Jacobs JL, Dinman JD (2008) PRFdb: a database of computationally predicted eukaryotic programmed -1 ribosomal frameshift signals. *BMC Genomics* 9(339):339.
8. Frankish A, et al. (2019) GENCODE reference annotation for the human and mouse genomes. *Nucleic Acids Res* 47(D1):D766–D773.
9. Szklarczyk D, et al. (2019) STRING v11: Protein-protein association networks with increased coverage, supporting functional discovery in genome-wide experimental datasets. *Nucleic Acids Res* 47(D1):D607–D613.

Supplementary figure S1

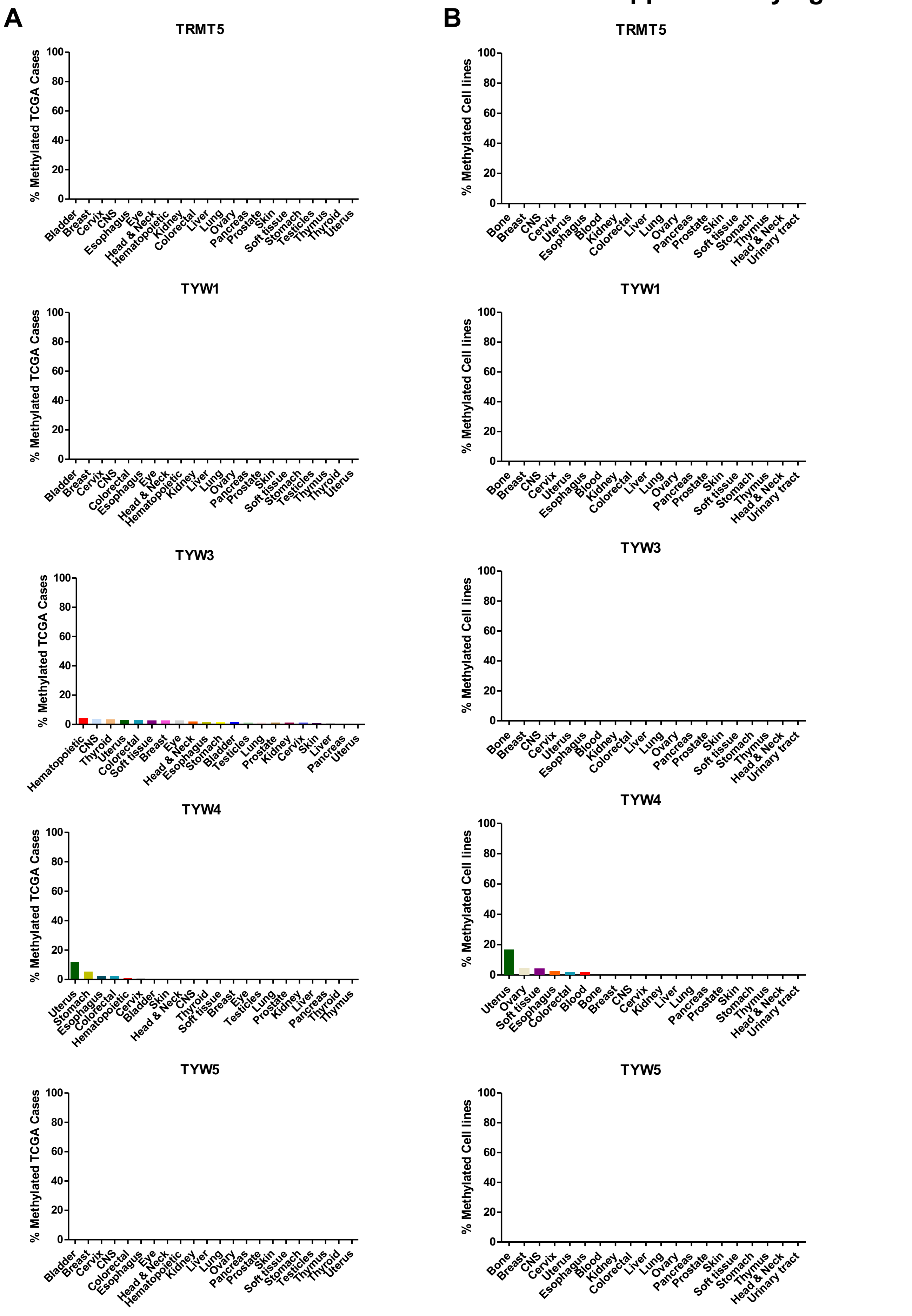

Supplementary figure S1. DNA methylation status of genes involved in OHyW and o2yW synthesis.

In silico screening of the percentage of methylated cases of TRMT5, TYW1, TYW3, TYW4 and TYW5 in (**A**) TCGA data sets of primary tumors and (**B**) in cancer cell lines.

Supplementary figure S2

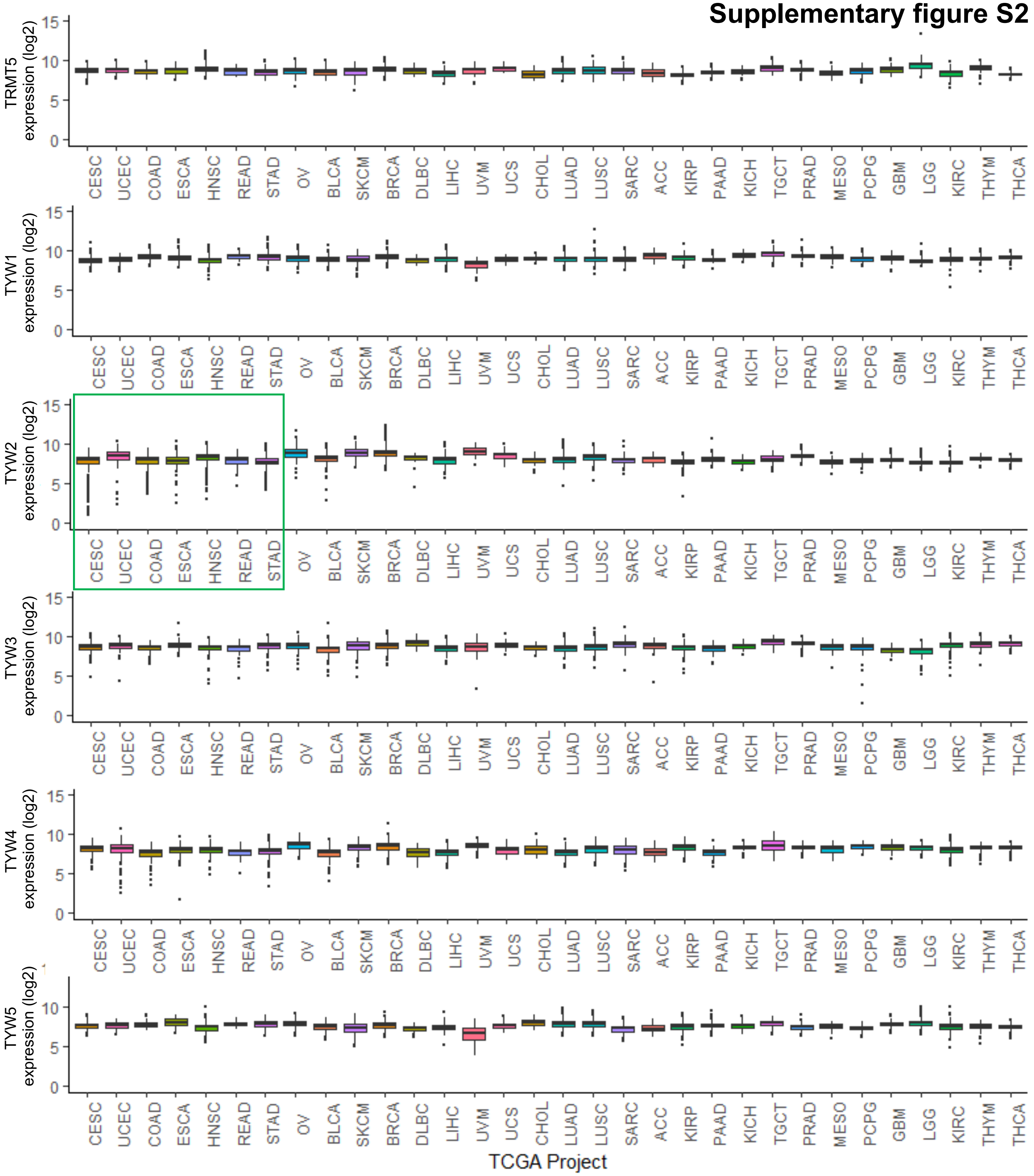

Supplementary figure S2. Expression levels of genes involved in OHyW and o2yW synthesis.

In silico screening of the expression of TRMT5, TYW1, TYW2, TYW3, TYW4 and TYW5 in TCGA data sets of primary tumors of the TCGA. Expression data is represented as the log2 of the RSEM-normalized values. For each transcript, TCGA projects are ordered from higher to lower TYW2 expression standard deviation. ACC, Adrenocortical carcinoma; BLCA, Bladder Urothelial Carcinoma; BRCA, Breast invasive carcinoma; CESC, Cervical squamous cell carcinoma and endocervical adenocarcinoma; CHOL, Cholangiocarcinoma; COAD, Colon adenocarcinoma; ESCA, Esophageal carcinoma; GBM, Glioblastoma multiforme; HNSC, Head and Neck squamous cell carcinoma; KICH, Kidney Chromophobe; KIRC, Kidney renal clear cell carcinoma; KIRP, Kidney renal papillary cell carcinoma; LGG, Brain Lower Grade Glioma; LIHC, Liver hepatocellular carcinoma; LUAD, Lung adenocarcinoma; LUSC, Lung squamous cell carcinoma; DLBC, Lymphoid Neoplasm Diffuse Large B-cell Lymphoma; MESO, Mesothelioma; OV, Ovarian serous cystadenocarcinoma; PAAD, Pancreatic adenocarcinoma; PCPG, Pheochromocytoma and Paraganglioma; PRAD, Prostate adenocarcinoma; READ, Rectum adenocarcinoma; SARC, Sarcoma; SKCM, Skin Cutaneous Melanoma; STAD, Stomach adenocarcinoma; TGCT, Testicular Germ Cell Tumors; THYM, Thymoma; THCA, Thyroid carcinoma; UCS, Uterine Carcinosarcoma; UCEC, Uterine Corpus Endometrial Carcinoma; UVM, Uveal Melanoma. Within a green square, the most disperse TCGA projects according to TYW2 expression correspond to the most methylated cancer types as seen in Fig. 1B.

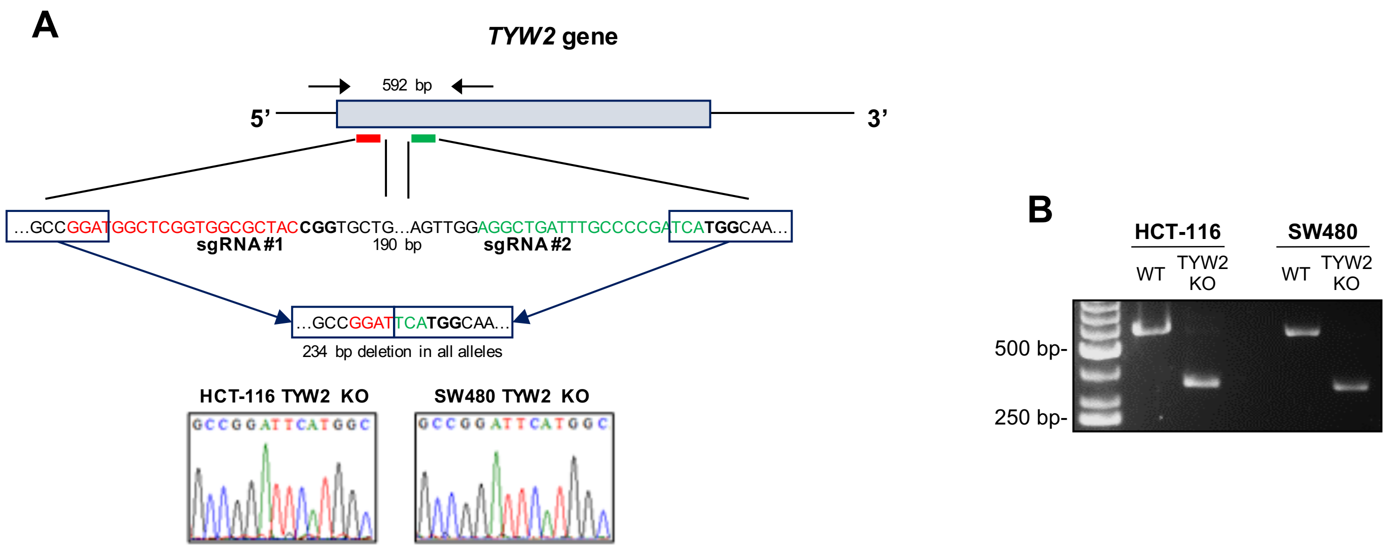

Supplementary figure S3. CRISPR/Cas9-mediated deletion in TYW2 gene body.

(**A**) Overview of the design of the CRISPR/Cas9 sgRNA to target TYW2. We used two different sgRNA (sequences are represented in red and green) separated 190 bp. Simultaneous transfection of the two sgRNA resulted in a deletion of 234 bp, shown by Sanger sequencing. Black arrows on the top of the gene represent the primers used to amplify this region. (**B**) Genomic PCR performed using primers flanking the CRISPR/Cas9 targeted region shows a deletion in TYW2 gene.

Supplementary figure S4

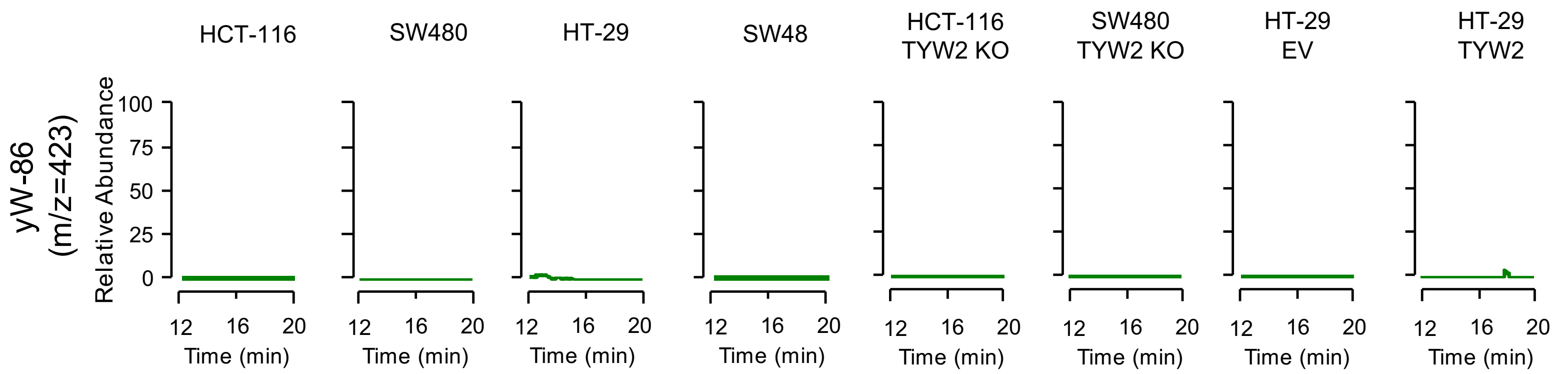

Supplementary figure S4. Hypomodified intermediary yW-86 is undetectable in $tRNA^{Phe}$ from colon cancer cell lines.

Nucleoside analysis of tRNAs by liquid chromatography-mass spectrometry (LC/MS) shows that the hypomodified intermediary yW-86 is absent in all the colon cancer cell lines and in all the derived models.

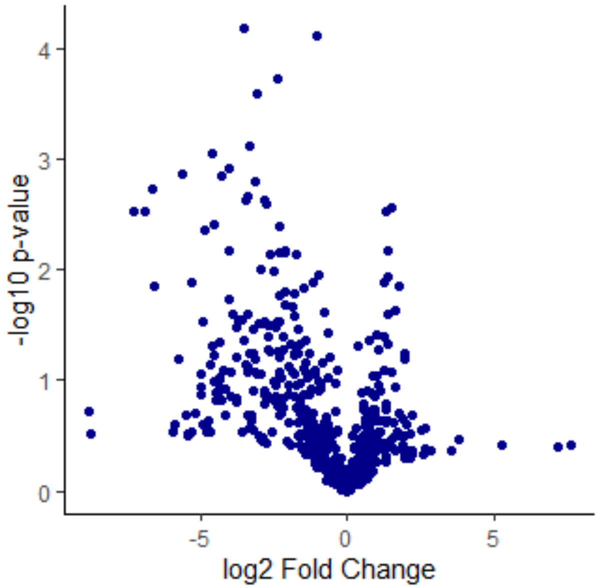

Supplementary figure S5. In silico expression analysis among 47 colon cancer cell lines of the transcripts that are downregulated in CRISPR/Cas9-mediated TYW2 depleted HCT-116 cell line.

Volcano plot summarizing the results of an in silico analysis of the expression of the 671 transcripts available from the 2046 downregulated in the RNA-seq experiment performed in HCT-116 cell line upon TYW2 depletion. Data shown represents the fold changed of expression of methylated cell lines (n=4) against unmethylated cell lines (n=43). 409 of these 671 transcripts (61%) were downregulated in the TYW2 hypermethylated/silenced cell lines in comparison to the TYW2 unmethylated/expressing cell lines. Data normality of the methylated subset of samples was assessed using a Shapiro-Wilk test. Statistical differences were calculated using two-tailed Student's t-test.

Supplementary figure S6

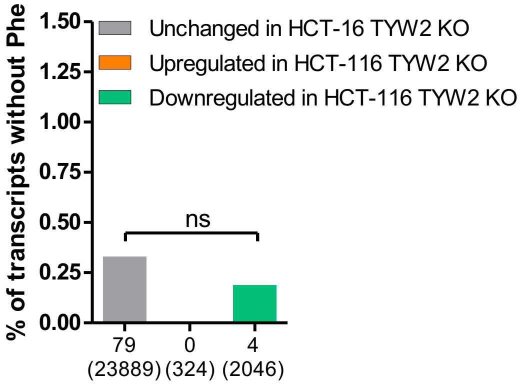

Supplementary figure S6. TYW2 loss does not affect transcripts that lack UUU and UUC codons (n=83).

The proportion of transcripts that do not encode for phenylalanine is unaltered among the unchanged genes and the deregulated ones in HCT-116 TYW2 KO compared to HCT-116 WT. The number of transcripts is provided below the corresponding bar. In brackets, total transcripts in each category. Statistical differences among proportions were calculated using a Chi-squared test. ns, no significant.

Supplementary figure S7

Number of significant transcripts in database: 661

Genes in the subnet: 407

PRF genes (red): 75

Other significant genes (green): 332

PPI enrichment p-value < 1e-20

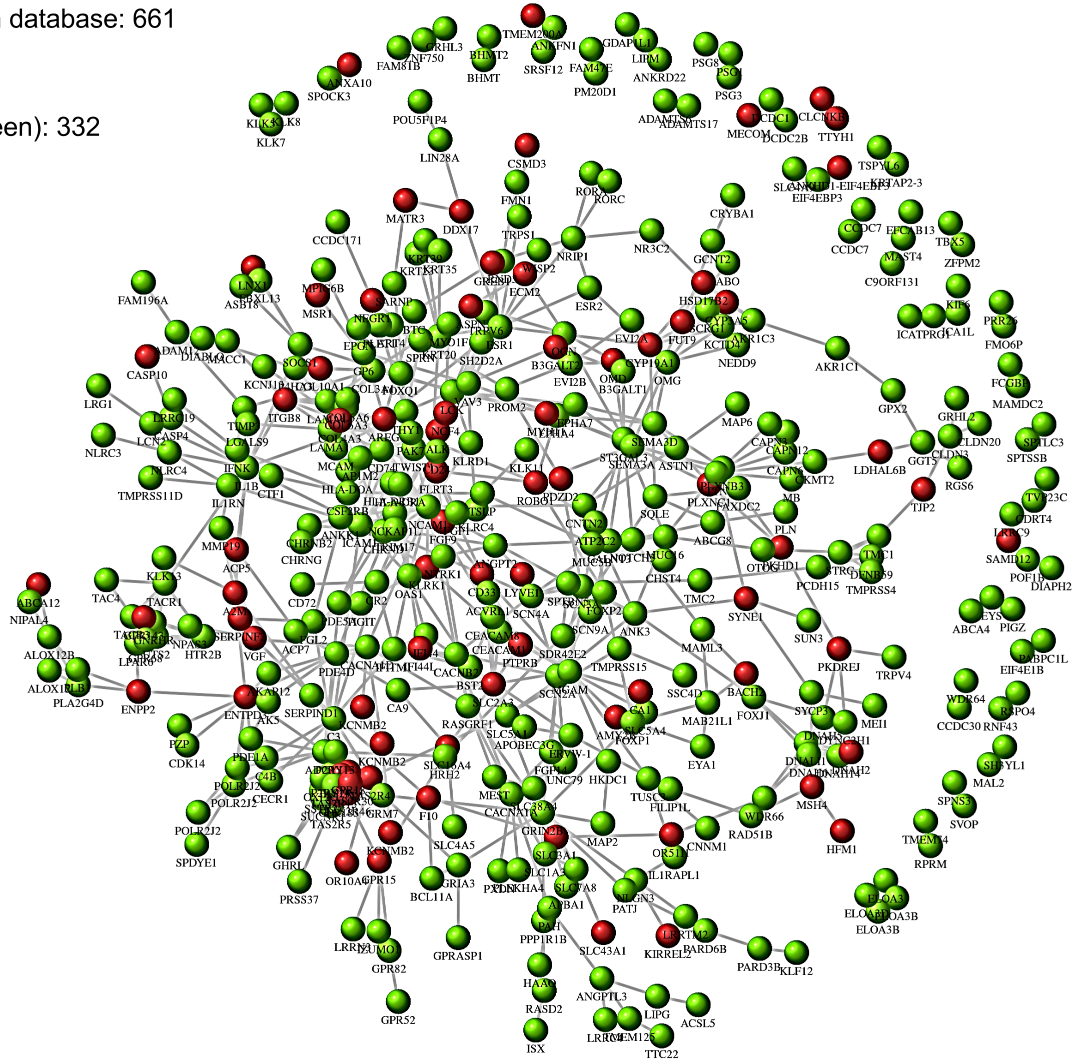

Supplementary figure S7. Network of predicted protein-protein interactions among the proteins that are downregulated in TYW2-depleted HCT-116 cells.

Network of predicted protein-protein interactions based on the STRING databse among the protein-coding transcripts that are downregulated in the RNA-sequencing experiment performed in CRISPR/Cas9-mediated TYW2 knockout HCT-116 cell line. In red, proteins that harbour at least one UUUU/C-based slippery sequences; in green, proteins that do not contain a UUUU/C-based slippery site. *p*-value corresponds to protein-protein interaction enrichment. Overal, we observed that 75 of the 109 PRF-proteins (68.8%) interacted with 332 proteins derived from the overall 661 coding downregulated transcripts (50.2%).

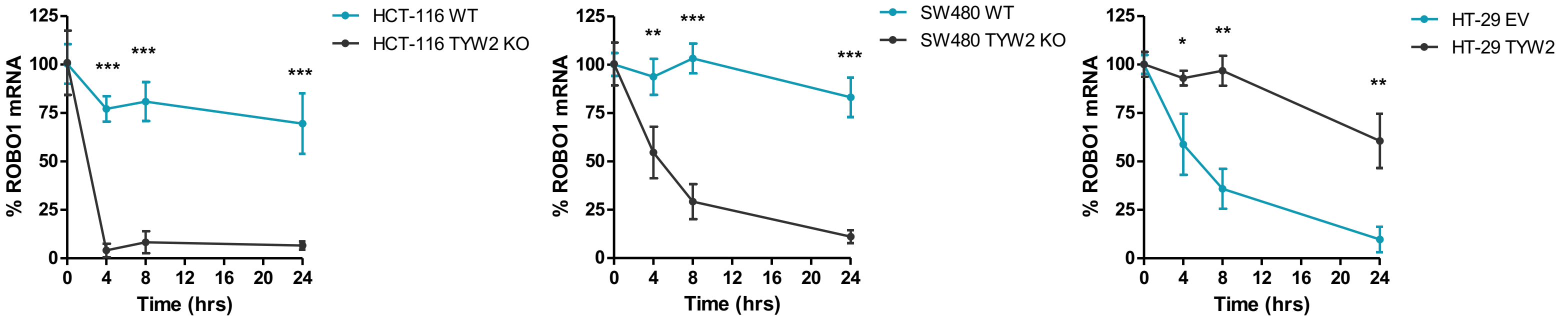

Supplementary figure S8. ROBO1 mRNA stability is decreased upon alpha-amanitin treatment in cells with TYW2 loss.

alpha-amanitin chase assay reveals a reduction in ROBO1 mRNA in CRISPR/Cas9-mediated deletion of TYW2 in HCT-116 and SW480 cells, but not in the wild-type cells. TYW2 transfection-mediated recovery in HT-29 cells results in a stabilization of ROBO1 transcript. Data shown represents the mean ± SD of three biological replicates and were analyzed using an unpaired two-tailed Student's t-test. * p<0.05; ** p<0.01; *** p<0.001.

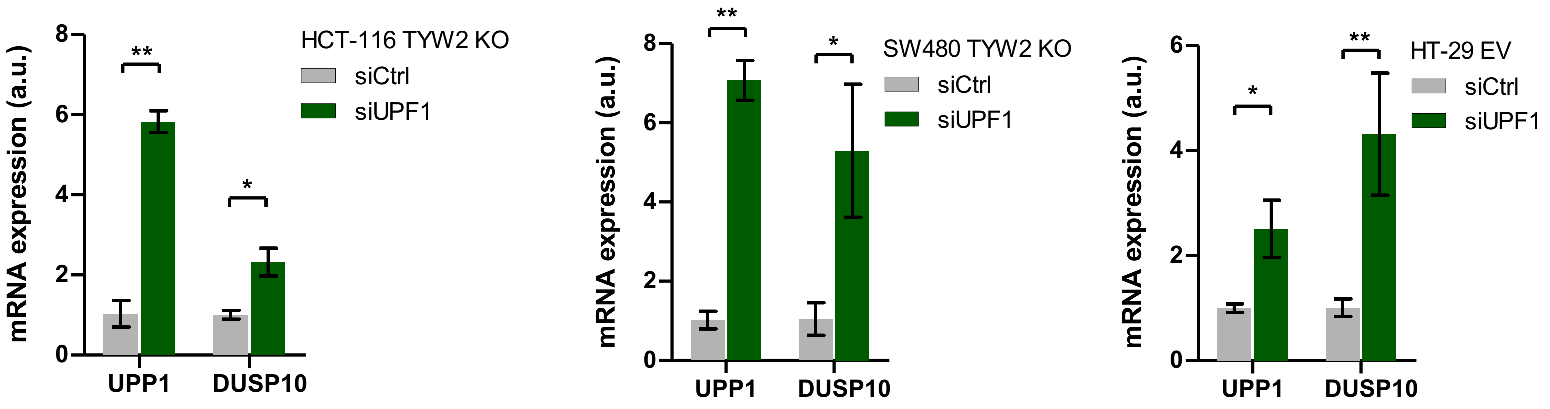

Supplementary figure S9. Known targets of nonsense-mediated decay (NMD) are upregulated upon UPF1 knockdown.

Nonsense-mediated mRNA decay inhibition using a siRNA against UPF1 in CRISPR/Cas9 TYW2-depleted HCT-116 and SW480 cells and in EV-transfected HT-29 cells results in an upregulation of two transcripts known to be degraded via UPF1. Data shown represents the mean ± SD of biological triplicates analyzed by unpaired two-tailed Student's t test. * $p < 0.05$; ** $p < 0.01$.

Supplementary figure S10

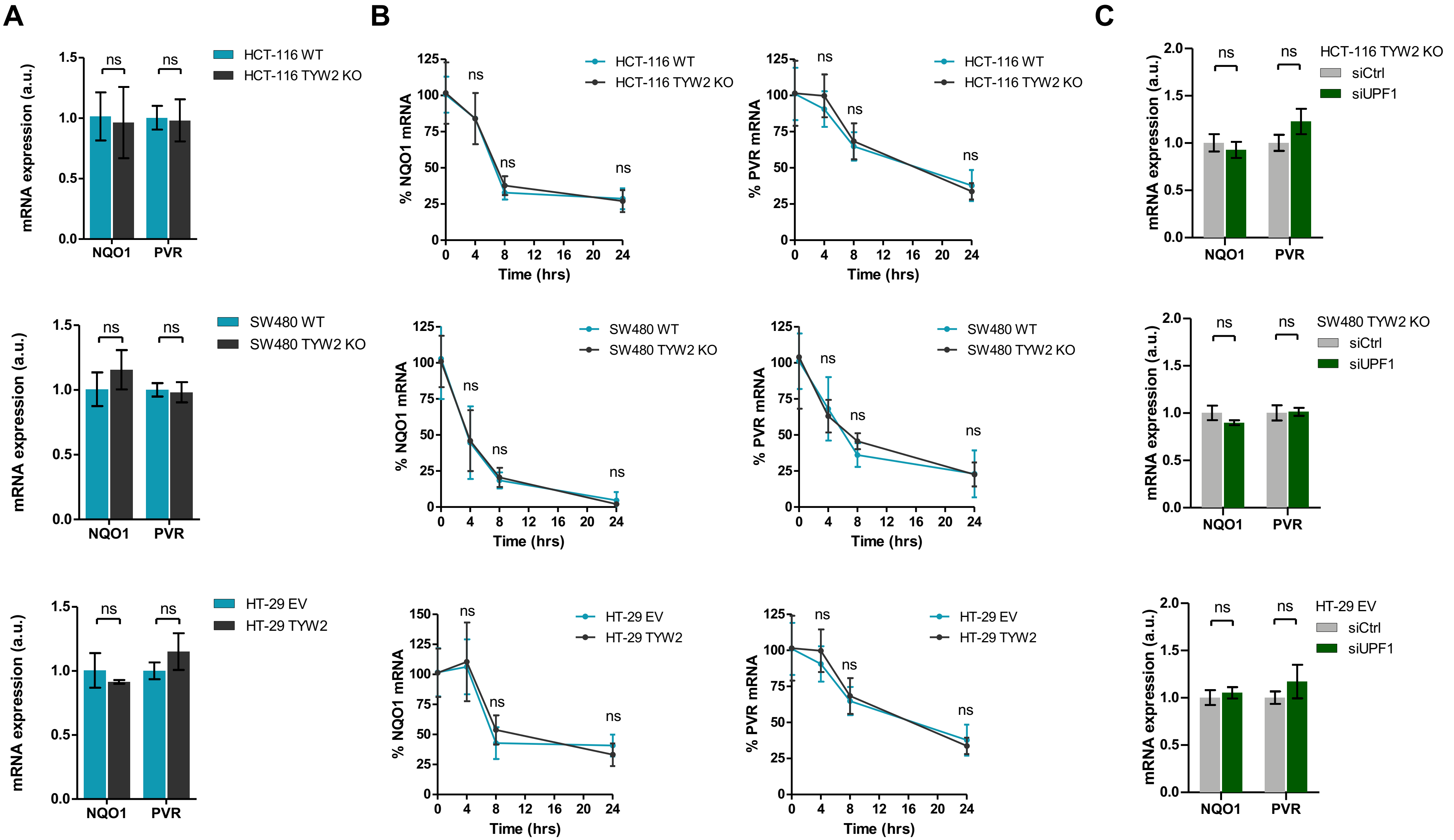

Supplementary figure S10. Genes without expression changes detected in the RNA-seq of TYW2 CRISPR/Cas9 deleted HCT-116 cells are not differentially degraded or accumulated upon nonsense-mediated mRNA decay (NMD) inhibition. The examples of the NQO1 and PVR genes.

(**A**) NQO1 and PVR mRNA levels in the studied cell line models determined by qRT-PCR are not affected by TYW2 expression levels. Data shown represents the mean ± SD of biological triplicates analyzed by unpaired two-tailed Student's t test. ns, not significant. (**B**) alpha-amanitin chase assay of NQO1 and PVR does not reveal any difference in mRNA stability between TYW2-silenced cells compared to their TYW2-expressing counterparts. For all cases, data shown represents the mean ± SD of biological triplicates analyzed by unpaired two-tailed Student's t test at each time point. ns, not significant. (**C**) Nonsense-mediated mRNA decay inhibition using a siRNA targeting UPF1 in TYW2-depleted cells does not affect NQO1 and PVR mRNA abundance. Data shown represents the mean ± SD of biological triplicates analyzed by unpaired two-tailed Student's t test. ns, not significant.

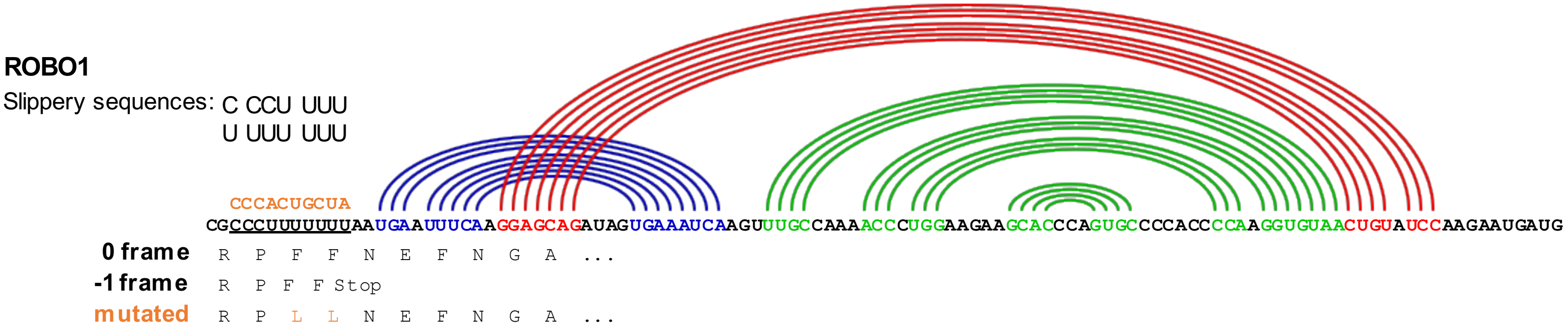

Supplementary figure S11. ROBO1 slippery sequence.

Schematic representation of the two consecutive slippery sequences identified in ROBO1 using PRFdb. The RNA sequences of different colors depict the putative folding of ROBO1 mRNA. This region of complicated secondary structure is preceded by the slippery heptamer where $tRNA^{Phe}$ recoils one position, represented as the underlined sequences containing UUUU/C. The amino acid sequence generated during translation is below the nucleotide sequence (0 frame). In case of -1 ribosome frameshifting event, a premature stop codon will appear and will originate a truncated protein. These sequences were cloned into the dual luciferase reporter as well as a mutant in which phenylalanine codons in the slippery heptamer are replaced by leucine codons in which -1 ribosome frameshift should not occur.

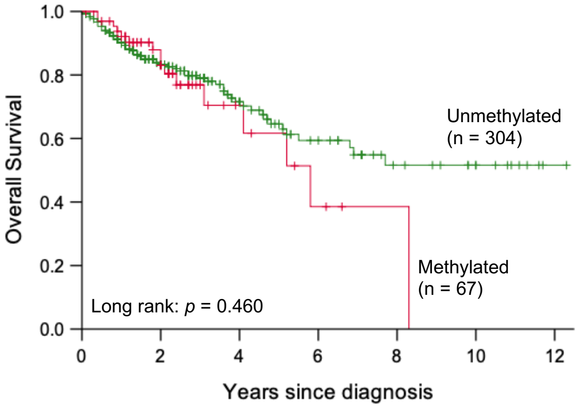

At risk:							TYW2
304	260	247	239	236	236	236	U
67	59	55	52	52			M

Supplementary figure S12. TYW2 DNA methylation status is not associated with patients' prognosis considering the complete cohort of TCGA colorectal cancer

Kaplan-Meier analyses of overall survival according to TYW2 methylation status in the TCGA COAD and READ projects considering the complete cohort of primary tumors. *p*-value corresponds to the log-rank test.

Supplementary figure S13

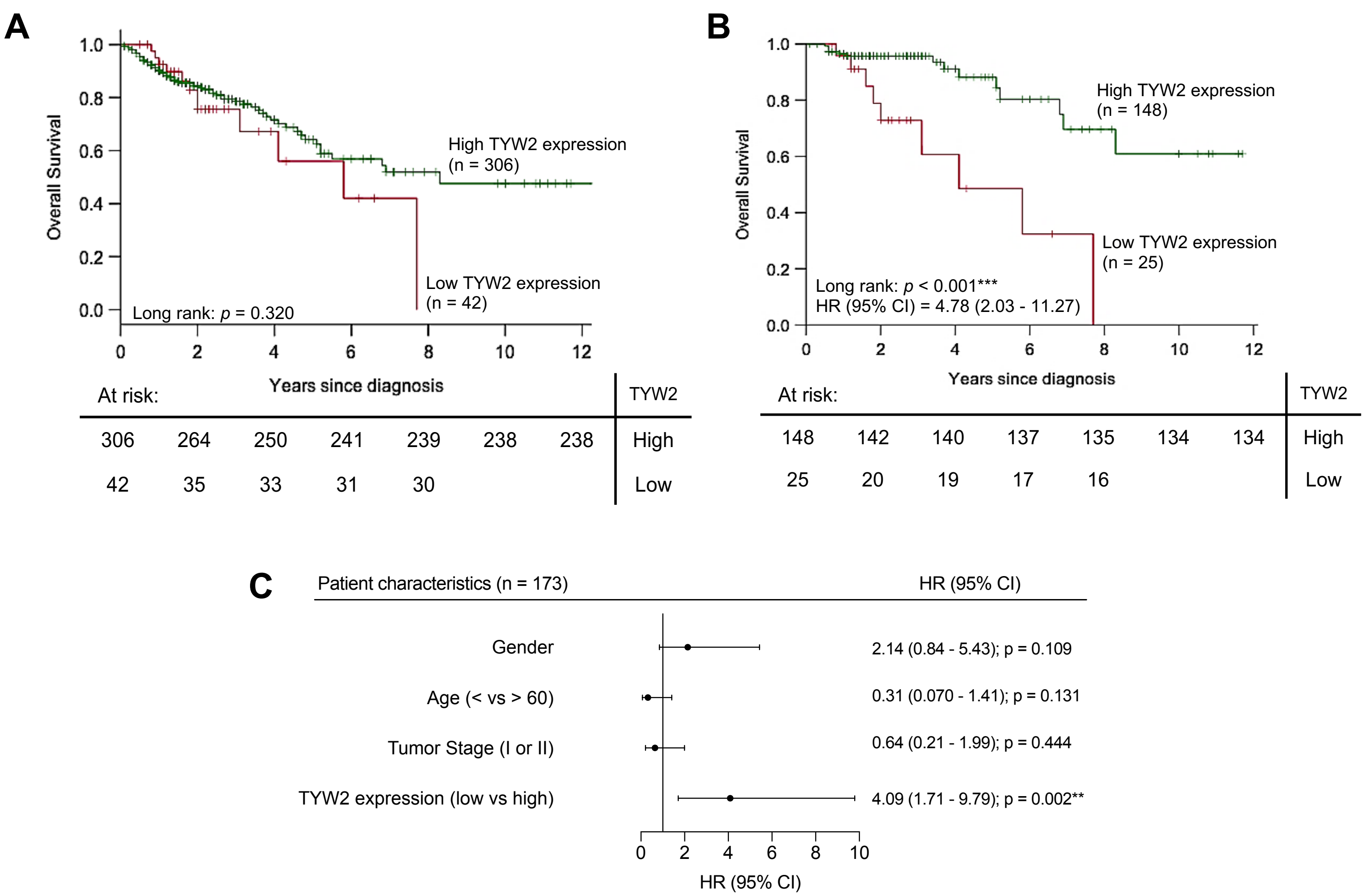

Supplementary figure S13. Low TYW2 expression is associated with shorter overall survival in patients with early-stage colorectal tumors.

Kaplan-Meier curve of overall survival according to the TCGA colorectal tumors in the complete cohort of primary tumors (**A**) and only in early-stage colorectal cancer patients (**B**) . Green line, high expression (log2 TYW2 expression > 7.0); red line, low expression (log2 TYW2 expression ≤ 7.0). *p*-value corresponds to log-rank test. ns, no significant, *** $p < 0.001$. (**C**) Forest plot representation of the Cox proportional hazard regression models shows that TYW2 reduced expression is an independent prognostic factor of poor overall survival in early-stage colorectal cancer patients (HR=4.09; 95% CI=1.71 to 9.79) HR, hazard ratio; CI, confidence interval. ns, not significant; ** $p < 0.001$.

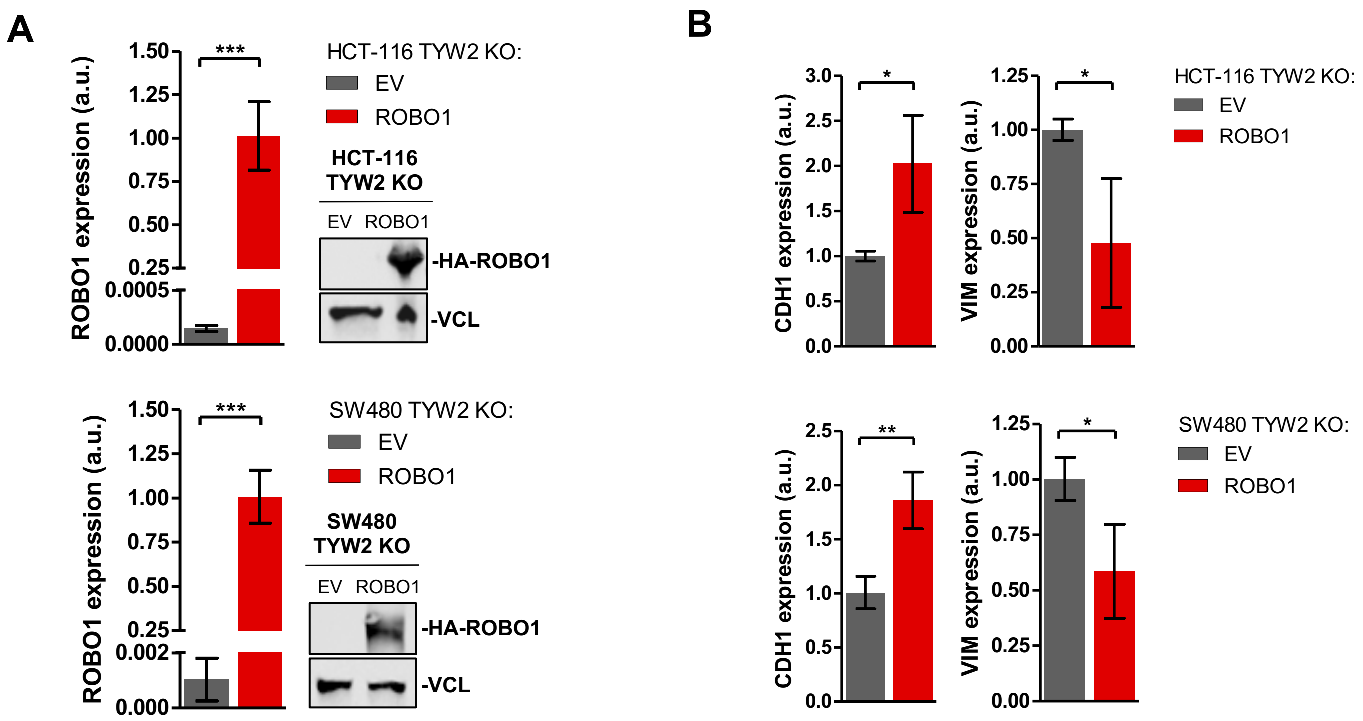

Supplementary figure S14. Rescue of ROBO1 expression in TYW2-silenced HCT-116 and SW480 cells reverts the epithelial-to-mesenchymal transition (EMT) phenotype.

(**A**) ROBO1 mRNA (*left*) and protein (*right*) levels in empty vector (EV) and ROBO1-transfected HCT-116 and SW480 TYW2 KO cell lines. Data shown represents the mean ± SD of three biological replicates and were analyzed using an unpaired two-tailed Student's t-test. *** $p<0.001$. (**B**) Assessment of EMT features by qRT-PCR of E-cadherin (CDH1) and vimentin (VIM) indicates that transfection-mediated recovery of ROBO1 reverts the mesenchymal features observed upon TYW2 CRISPR-Cas9 deletion. Data shown represents the mean ± SD of three biological replicates and were analyzed using an unpaired two-tailed Student's t-test. * $p<0.05$; ** $p<0.01$.

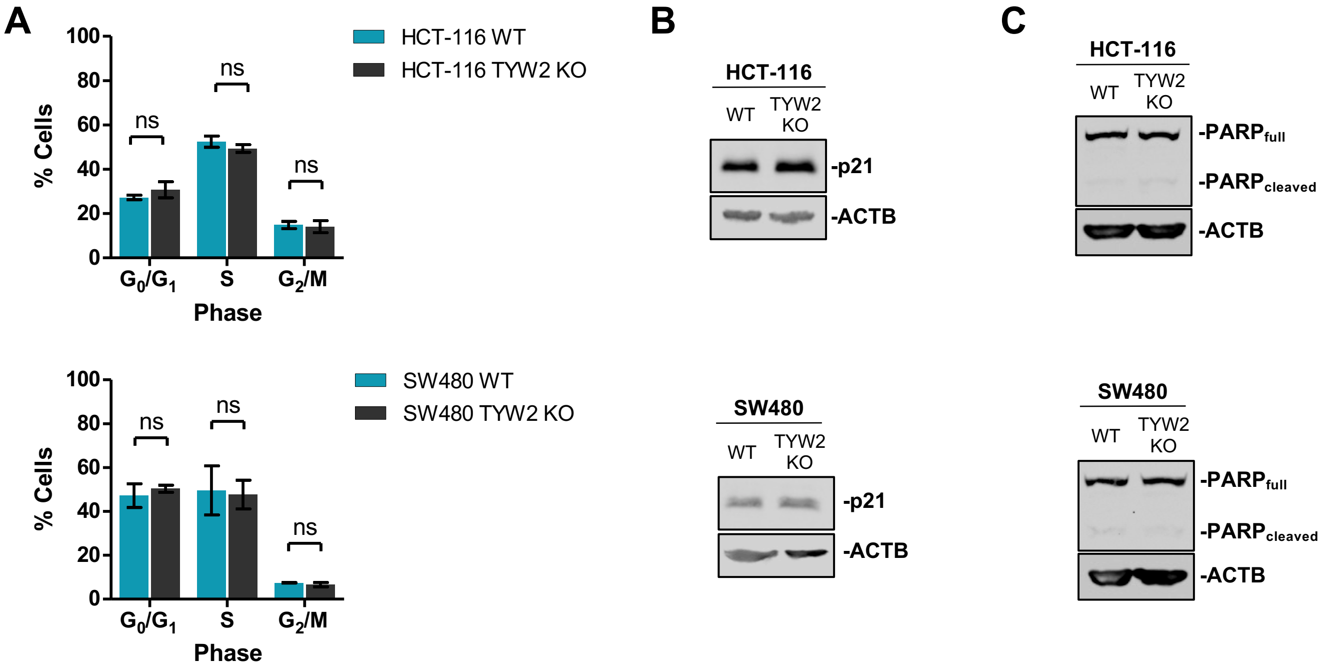

Supplementary figure S15. TYW2 silencing does not affect cell cycle, arrest and death.

(**A**) Cell cycle analysis, assessed by BrdU and 7AAD incorporation, does not show any differences between the unmethylated HCT-116 and SW480 cell lines and their CRISPR/Cas9-mediated TYW2 knock-out counterparts. Data shown represents the mean ± SD of biological triplicates analyzed by unpaired two-tailed Student's t test. ns, no significant. (**B**) Similar protein levels of p21 determined by western-blot indicate no differences in cell cycle arrest. (**C**) PARP cleavage evaluated by western-blot indicates no differences in cell death between TYW2-expressing (WT) and TYW2 knock-out (KO) cell lines.

Supplementary Table S1. List of the 109 downregulated genes in TYW2-depleted HCT-116 cell line that contains at least one predicted slippery site composed of UUUU/C by the PRFdb tool. Genes are ordered according to the log_2 fold change obtained in the RNA-seq experiment.

Gene Symbol	log2 fold change	Adjusted p-value	# of sites	Predicted slippery sequences
KCNH8	-7.20	5.56E-05	3	GGGUUUC, GGGUUUU, AAAUUUC
IFI44L	-7.02	0.00091337	2	UUUUUUA, UUUUUUC
FLRT3	-6.57	0.00517664	1	GGGUUUC
MSR1	-6.39	0.00249354	1	AAAUUUC
GPR171	-6.32	0.00669546	2	UUUUUUU, AAAUUUC
GPR22	-5.17	5.09E-05	3	UUUUUUA, AAAUUUU, UUUUUUC
NEGR1	-4.98	0.01293809	1	AAAUUUU
TAS2R30	-4.87	0.02262428	1	AAAUUUU
GPR18	-4.69	0.00315055	2	CCCUUUU, UUUUUUC
EHF	-4.44	3.11E-62	1	CCCUUUC
OR10A4	-4.41	0.02979729	1	CCCUUUU
PKHD1	-4.38	0.0169595	1	UUUUUUU
OR2D3	-4.37	0.03957446	2	UUUUUUU, UUUUUUC
ALK	-4.35	0.00049522	3	CCCUUUC, GGGUUUC, GGGUUUU
A2M	-4.32	4.89E-07	1	CCCUUUC
B3GALT2	-4.30	0.01196415	1	UUUUUUA
ADAM20	-4.21	0.00105178	1	UUUUUUA
ASPN	-4.19	0.00581219	1	UUUUUUU
ROBO1	-4.16	0.000992	2	CCCUUUU, UUUUUUU
HSD17B2	-4.14	0.00635917	1	CCCUUUU
SFRP5	-4.05	0.00554296	1	CCCUUUC
CA1	-4.02	0.00052328	1	AAAUUUU
MECOM	-4.00	7.58E-10	1	AAAUUUC
COL10A1	-3.88	8.42E-05	1	GGGUUUU
OMD	-3.68	0.01350182	2	UUUUUUA, GGGUUUU
FUT9	-3.63	0.00120964	1	AAAUUUU
TAS2R46	-3.60	0.00523273	1	AAAUUUU
OR52D1	-3.50	0.02026787	1	CCCUUUC
ENPP2	-3.49	0.00661371	1	CCCUUUC
ECM2	-3.48	0.00214317	1	UUUUUUU
CSMD3	-3.39	0.00546975	4	UUUUUUA, UUUUUUU, AAAUUUU, AAAUUUC
C17orf78	-3.33	0.00751464	1	UUUUUUA
CD33	-3.17	1.85E-12	1	AAAUUUC
LYVE1	-3.12	0.00246607	1	CCCUUUU

ACP5	-3.09	0.00155529	1	CCCUUUC
GPR15	-3.07	2.51E-06	1	CCCUUUC
OR51I1	-2.89	0.00387208	2	CCCUUUC, CCCUUUU
ENTPD3	-2.87	0.04570855	1	AAAUUUC
SLC3A1	-2.85	0.02432329	1	AAAUUUC
GPR34	-2.84	0.03640931	1	CCCUUUC
NCF4	-2.84	0.02200344	1	CCCUUUC
OR2AG2	-2.78	0.01189788	1	CCCUUUC
GPR183	-2.70	0.00549392	1	CCCUUUU
TAS2R31	-2.68	0.01882221	1	AAAUUUU
C9orf50	-2.60	0.00307219	1	CCCUUUC
LDHAL6B	-2.54	0.03136972	1	CCCUUUC
STK32A	-2.42	0.02134328	1	CCCUUUC
DNAH2	-2.41	4.39E-08	2	CCCUUUU, UUUUUUU
CYP19A1	-2.41	0.00077977	1	AAAUUUU
ECT2L	-2.34	0.00258119	2	UUUUUUA, AAAUUUU
F10	-2.31	1.15E-05	1	GGGUUUC
ANGPTL1	-2.27	0.02370045	1	CCCUUUC
NEXN	-2.19	0.006749	1	AAAUUUU
ZNF660	-2.18	0.0320315	1	AAAUUUC
CLCNKB	-2.12	0.00091337	1	CCCUUUC
SAMD3	-2.10	0.000665	1	CCCUUUC
TAS2R4	-2.05	0.00314183	2	UUUUUUU, AAAUUUU
SLC2A3	-2.01	9.39E-06	1	GGGUUUU
ANXA10	-1.96	1.85E-05	1	AAAUUUU
TSHZ2	-1.96	0.01663322	1	GGGUUUC
VGF	-1.96	1.92E-12	1	CCCUUUC
RAD51AP2	-1.93	0.00325097	3	CCCUUUU, AAAUUUU, UUUUUUU
KCNMB2	-1.89	0.04518552	1	AAAUUUU
ABI3BP	-1.88	0.03443561	2	CCCUUUU, GGGUUUC
CASP10	-1.85	9.01E-07	1	CCCUUUC
OR51B2	-1.82	0.00029773	2	CCCUUUU, GGGUUUU
KLRK1	-1.78	0.00372001	1	UUUUUUC
HFM1	-1.73	0.00632873	2	UUUUUUU, AAAUUUU
MYH11	-1.72	0.02123549	1	GGGUUUC
GREB1	-1.72	2.64E-09	1	CCCUUUC
KIRREL2	-1.69	0.00811129	1	CCCUUUC
ELOVL2	-1.67	0.0139305	1	AAAUUUU
FGF9	-1.66	0.04705264	1	UUUUUUA
DDX17	-1.66	5.72E-18	1	AAAUUUU

PTPRB	-1.65	0.00063183	1	UUUUUUC
TMEM200 A	-1.64	3.03E-06	2	UUUUUUU, GGGUUUU
MATR3	-1.64	0.00294423	1	UUUUUUU
TJP2	-1.63	0.0461731	1	UUUUUUC
SLC16A4	-1.61	0.01882439	2	UUUUUUU, UUUUUUC
PLA2R1	-1.60	0.00012883	2	AAAUUUU, AAAUUUC
TTYH1	-1.56	0.02610305	2	CCCUUUC, AAAUUUC
AMY2B	-1.52	0.00044065	2	CCCUUUC, GGGUUUC
TMPRSS1 3	-1.51	0.01606597	1	GGGUUUC
KIAA0825	-1.48	0.02337849	1	AAAUUUU
SLCO6A1	-1.48	0.03444698	1	UUUUUUU
PDZD2	-1.40	0.01771051	1	AAAUUUU
LNX1	-1.39	0.00334108	2	UUUUUUA, UUUUUUC
ITGB8	-1.38	3.11E-06	2	UUUUUUU, CCCUUUU
PLXNC1	-1.35	0.02045559	1	AAAUUUU
ZNF84	-1.31	4.00E-05	2	UUUUUUU, AAAUUUC
MUM1L1	-1.30	0.01059227	1	AAAUUUU
PKDREJ	-1.30	0.00839977	5	GGGUUUC, UUUUUUG, UUUUUUC, UUUUUUA, UUUUUUU
SAMD12	-1.26	1.91E-06	1	AAAUUUC
TBC1D19	-1.25	0.01824425	2	CCCUUUU, UUUUUUC
KIAA1257	-1.22	0.01942908	1	AAAUUUC
MSH4	-1.19	0.01239947	2	AAAUUUU, AAAUUUC
AREG	-1.18	0.01306412	1	AAAUUUC
BACH2	-1.16	0.00085021	2	CCCUUUC, CCCUUUU
SYNE1	-1.15	0.02200093	5	GGGUUUC, AAAUUUC, GGGUUUU, UUUUUUU, AAAUUUU
TMC4	-1.12	0.00015592	1	UUUUUUC
SLC43A1	-1.11	0.0098746	1	GGGUUUU
ABCA12	-1.10	0.04307432	2	CCCUUUU, AAAUUUC
CYP3A5	-1.10	0.01824029	2	UUUUUUA, AAAUUUU
RASIP1	-1.09	0.01599997	1	CCCUUUC
ANKHD1-EIF4EBP3	-1.08	0.03040577	1	AAAUUUU
COL4A3	-1.03	0.00013628	2	GGGUUUC, GGGUUUU
GPR143	-1.03	0.03453523	2	GGGUUUU, AAAUUUU
TRIM15	-1.02	0.00753697	1	CCCUUUC
SERPINF2	-1.01	0.02897758	1	GGGUUUC

Supplementary Table S2. Detailed list of antibodies and reagents, commercial assays, primers and vectors used, together with their commercial reference.

KEY RESOURCES TABLE

REAGENT or RESOURCE	SOURCE	IDENTIFIER
**Antibodies**		
anti-TYW2	Novus Bio	NBP1-76583
anti-ROBO1	Abcam	ab7279
anti-Calnexin	Cell Signaling	2679
anti-HA HRP-conjugated	Sigma	H6533
anti-V5	Cell Signaling	13202
anti-PARP	Cell Signaling	9542
anti-p21	Cell Signaling	2497
anti-β-Actin HRP-conjugated	Sigma	A3854
anti-LaminB1	Abcam	ab16048
anti-Vinculin HRP-conjugated	Cell Signaling	18799
anti-rabbit HRP-conjugated secondary antibody	Sigma	A0545
**Bacterial and Virus Strains**		
E. coli DH5α	Thermofisher	18265017
**Chemicals and Critical Commercial Assays**		
5'-azacytidine	Sigma	A2385
Sulforhodamine B	Sigma	S1402
Trichloroacetic Acid	Sigma	T4885
JetPrime Transfection Reagent	Polyplus Transfection	114-75
SYBR Green PCR Master Mix	Life Technologies	4312704
Proteinase K Recombinant PCR grade	Thermofisher Scientific	EO0492
Actinomycin D	Sigma	A4262
TRIzol reagent	Thermofisher Scientific	15596026
alpha-amanitin	MedChemExpress	HY-19610
Nuclease T_1	FUJIFILM Wako Pure Chemical	145-08221
Phosphodiesterase I	Worthington Biochemical	LS003926
Alkaline phosphatase (*E. coli* C75)	Takara Bio	2120A
EZ-DNA methylation Gold Kit	Zimo research	D5006
Nucleospin Gel and PCR Clean-up	Macherey Nagel	740609.250
Nucleospin 96 plasmid	Macherey Nagel	740625.24
Maxwell RSC simply RNA tissue	Promega	AS1340
RevertAid First Strand cDNA synthesis kit	Thermofisher Scientific	K1622
Dual Glo Luciferase Assay System	Promega	E2920
6.5mm Transwell with 8.0µm pore polycarbonate membrane insert	Corning	3422
APC BrdU Flow kit	BD Biosciences	552598
pGEM-T Easy Vector	Promega	A1360
ProteoExtract® Subcellular Proteome Extraction Kit	Merck Millipore	539790
**Cell Lines**		
HCT-116	ATCC	CCL-247
HT-29	ATCC	HTB-38
SW48	ATCC	CCL-231
SW480	ATCC	CCL-228
HEK-293	ATCC	CRL-1573

Oligonucleotides			
BSP_TYW2_Fd	ATGTTTTTTTAGGTTGAAAAAAAAG	This study	-
BSP_TYW2_Rv	AAACCAAAACTCAATCACAACT	This study	-
qPCR_TYW2_Fd	CCTGCCCAAAAATTGTGTCT	This study	-
qPCR_TYW2_Rv	AGAGTTCCGGTCCCAGATTT	This study	-
qPCR_ROBO1_Fd	GGAGTCAGGGGCACAAGAAA	This study	-
qPCR_ROBO1_Rv	GGCCTCGTTCATCTTCCTCC	This study	-
qPCR_VIM_Fd	CTTAAAGGAACCAATGAGTCCCT	This study	-
qPCR_VIM_Rv	AGTGAATCCAGATTAGTTTCCCTC	This study	-
qPCR_CDH1_Fd	GGGGTCTGTCATGGAAGGTG	This study	-
qPCR_CDH1_Rv	GAAACTCTCTCGGTCCAGCC	This study	-
qPCR_GAPDH_Fd	GAAGGTGAAGGTCGGAGTC	This study	-
qPCR_GAPDH_Rv	TGGACTCCACGACGTACTCA	This study	-
qPCR_UPF1_Fd	CCATCCCCTTCAACCTGGTC	This study	-
qPCR_UPF1_Rv	GTTGGGGAGGTTAGTCTGGC	This study	-
qPCR_PVR_Fd	CTACACCTGCCTGTTCGTCA	This study	-
qPCR_PVR_Rv	GGTCTGAGTGCCAGGTGATT	This study	-
qPCR_NQO1_Fd	AAAGGACCCTTCCGGAGTAA	This study	-
qPCR_NQO1_Rv	CCATCCTTCCAGGATTTGAA	This study	-
qPCR_UPP1_Fd	CAGAGCAGGCAGTGGATACC	This study	-
qPCR_UPP1_Rv	CTGCTTGTCCTTCTCCGTGT	This study	-
qPCR_DUSP10_Fd	GCGAGTCCATAGCTGAAGAGG	This study	-
qPCR_DUSP10_Rv	GATGACAGGAGGGTGGCTG	This study	-
qPCR_28S_Fd	CAGGGGAATCCGACTGTTTA	This study	-
qPCR_28S_Rv	ATGACGAGGCATTTGGCTAC	This study	-
TYW2_sgRNA1_Fd	CACCGGTAGCGCCACCGAGCCATC	This study	-
TYW2_sgRNA1_Rv	AAACGATGGCTCGGTGGCGCTACC	This study	-
TYW2_sgRNA2_Fd	CACCGAGGCTGATTTGCCCCGATCA	This study	-
TYW2_sgRNA2_Rv	AAACTGATCGGGGCAAATCAGCCTC	This study	-
TYW2-KO_seq_Fd	TGTGGTTGTTAGCAACATGGA	This study	-
TYW2-KO_seq_Rv	CTCTACCCAGCCATGGTCAC	This study	-
siRNA_UPF1		Thermofisher Scientific	ID:12990
siRNA_Negative_Control_1		Thermofisher Scientific	AM4611
Cloning_TYW2_Fd	AAAAAAGAATTCGCCGCCACCATGAGAG AGAATGTGGTTGTTAGCAACATGGAGAG AGAAAGTGGGAAGCCCGTGGCTGT	This study	-
Cloning_TYW2_Rv	AAAAAAGCGGCCGCCTACTTATCGTCGT CATCCTTGTAATCGCCGGAGCCGCCAAC TGAAGGACAGGGGCAGCATTCCAGATCC AGGACTATGTGATCCACATGGGG	This study	-
PRF_HindIII-RLuc_Fd	AAAAAAAAGCTTGCCGCCACCATGTACC CCTACGACGTGCCCGACTACGCCGGATC AGGAGCTTCCAAGGTGTACGACCCCGAG CAACGCAAAC	This study	-
PRF_EcoRI-RLuc_Rv	TTTTTTGCGGCCGCGAATTCTCCTGATCC CTGCTCGTTCTTCAGCACGCGCTCCACG AAG	This study	-
PRF_KpnI-FLuc_Fd	AAAAAAGCGGCCGCGAATTCGGTACCGG ATCAGGAGCCGATGCTAAGAACATTAAG AAGGGCCCTGCTCCCTTCTACC	This study	-
PRF_XhoI-FLuc_Rv	TTTTTTCTCGAGTTACACGGCGATCTTGC CGCCTTTCTTAGCCTTGATCAGGATCTC	This study	-
PRF_HIV_Fd	AATTCAATTTTTTAGGGAAGATCTGGCCT TCCCACAAGGGAAGGCCAGGGAATTTTC TTCAGGGTAC	This study	-

PRF_HIV_Rv	CCTGAAGAAAATTCCCTGGCCTTCCCTTGTGGGAAGGCCAGATCTTCCCTAAAAAATTG	This study	-
PRF_ROBO1_Fd	AAAAAAAGAATTCCGCCCTTTTTTTAATGAATTTCAAGGAGCAGATAGTGAAATCAAGTTTGCCAAAACCCTGGAAGA	This study	-
PRF_ROBO1_Rv	TTTTTTTGGTACCTCCATCATTCTTGGATACAGTTACACCTTGGGGTGGGGCACTGGGTGCTTCTTCCAGGGTTTTGG	This study	-
PRF_ROBO1mut_Fd	AAAAAAAGAATTCCGCCCACTGCTAAATGAATTTCAAGGAGCAGATAGTGAAATCAAGTTTGCCAAAACCCTGGAAGA	This study	-
PRF_EcoRI-HA-GST1_Fd	AAAAAAAAGAATTCGCCGCCACCATGTACCCCTACGACGTGCCCGACTACGCCGGATCAGGATCCCCTATACT	This study	-
PRF_NotI-GST2_Rv	TTTTTTTGCGGCCGCTTAATGATGATGATGATGATGTCCTGATCCCAGATCCGATTTTGGAGGATGGTCGCC	This study	-
PRF_AgeI-GST2_Fd	AAAAAAAAACCGGTCAGGAGGATCCCCTATACTAGGTTATTGGAAAATTAAGGGCCTTGT	This study	-
PRF_NheI-GST1_Rv	AAAAAAAAGCTAGCCAGATCCGATTTTGGAGGATGGTCGCC	This study	-
PRF_NheI-HIV-Fd	AAAAAAAAGCTAGCGGCTGTTGGAAATGTGGAAAGGAAGGACAC	This study	-
PRF_AgeI-HIV_Rv	AAAAAAAACCGGTTTATGGGGCTGTTGGCTCTGGTCTGCTCT	This study	-
Cloning_ ROBO1_Fd	AAAAAAAAGCTTCTCGAGGCCGCCACCATGATTGCGGAGCCCGCTCACTTTTACCTGTTTGGATTAATATGTCTCTGTTCAG	This study	-
Cloning_ROBO1_Rv	TTTTTTGCGGCCGCTCAGGCGTAGTCGGGCACGTCGTAGGGGTATCCTGATCCGCTTTCAGTTTCCTCTAATTCTTCATTATTATCTTCTCCTCTTTCATATCCTCCAAGTACCTGCATTTCTGCAATATTTCTTCGACCT	This study	-
**Vectors**			
psPax2		Addgene	12260
pMD2.G		Addgene	12259
pLVX-IRES-ZsGreen1		Clonetech	632187
pSpCas9(BB)-2A-GFP		Addgene	48138
pcDNA4 T/O		Thermofisher Scientific	V102020
**Software and Algorithms**			
Bioedit		Bio-Soft	
GraphPad Prism 5		GraphPad Software, Inc	
SPSS		IBM	
Methyl Primer Express v1.0		Applied Biosystems	
UCSC Genome Browser		UCSC	
ImageJ		NCI	
Primer3		Source Forge	
R Programming Bioconductor packages		R project	
